# Hsa_circ_0003258 promotes prostate cancer metastasis by complexing with IGF2BP3 and sponging miR-653-5p

**DOI:** 10.1186/s12943-021-01480-x

**Published:** 2022-01-05

**Authors:** Yu-Zhong Yu, Dao-Jun Lv, Chong Wang, Xian-Lu Song, Tao Xie, Tao Wang, Zhi-Min Li, Jia-Ding Guo, Du-Jiang Fu, Kang-Jin Li, Ding-Lan Wu, Franky Leung Chan, Ning-Han Feng, Zhe-Sheng Chen, Shan-Chao Zhao

**Affiliations:** 1 Department of Urology, Nanfang Hospital, Southern Medical University, Guangzhou, 510515 China; 2 Department of Urology, the Third Affiliated Hospital of Southern Medical University, Guangzhou, 510500 China; 3 Department of Urology, the Third Affiliated Hospital of Guangzhou Medical University, Guangzhou, 510150 China; 4 Department of Radiotherapy, Affiliated Cancer Hospital & Institute of Guangzhou Medical University, Guangzhou, 510095 China; 5 Shenzhen Key Laboratory of Viral Oncology, The Clinical Innovation & Research Center (CIRC), Shenzhen Hospital, Southern Medical University, Shenzhen, 518110 China; 6 School of Biomedical Sciences, The Chinese University of Hong Kong, Hong Kong, 999077 China; 7 Department of Urology, Affiliated Wuxi No. 2 Hospital, Nanjing Medical University, Wuxi, 214002 China; 8 Department of Pharmaceutical Sciences, College of Pharmacy and Health Sciences, St. John’s University, Queens, New York, NY 11439 USA

**Keywords:** Prostate cancer, EMT, Hsa_circ_0003258, MiR-653-5p, IGF2BP3

## Abstract

**Background:**

More and more studies have shown that circular RNAs (circRNAs) play a critical regulatory role in many cancers. However, the potential molecular mechanism of circRNAs in prostate cancer (PCa) remains largely unknown.

**Methods:**

Differentially expressed circRNAs were identified by RNA sequencing. The expression of hsa_circ_0003258 was evaluated using quantitative real-time PCR and RNA in situ hybridization. The impacts of hsa_circ_0003258 on the metastasis of PCa cells were investigated by a series of in vitro and in vivo assays. Lastly, the underlying mechanism of hsa_circ_0003258 was revealed by Western blot, biotin-labeled RNA pulldown, RNA immunoprecipitation, luciferase assays and rescue experiments.

**Results:**

Increased expression of hsa_circ_0003258 was found in PCa tissues and was associated with advanced TNM stage and ISUP grade. Overexpression of hsa_circ_0003258 promoted PCa cell migration by inducing epithelial mesenchymal transformation (EMT) in vitro as well as tumor metastasis in vivo*,* while knockdown of hsa_circ_0003258 exerts the opposite effect. Mechanistically, hsa_circ_0003258 could elevate the expression of Rho GTPase activating protein 5 (ARHGAP5) via sponging miR-653-5p. In addition, hsa_circ_0003258 physically binds to insulin like growth factor 2 mRNA binding protein 3 (IGF2BP3) in the cytoplasm and enhanced HDAC4 mRNA stability, in which it activates ERK signalling pathway, then triggers EMT programming and finally accelerates the metastasis of PCa.

**Conclusions:**

Upregulation of hsa_circ_0003258 drives tumor progression through both hsa_circ_0003258/miR-653-5p/ARHGAP5 axis and hsa_circ_0003258/IGF2BP3 /HDAC4 axis. Hsa_circ_0003258 may act as a promising biomarker for metastasis of PCa and an attractive target for PCa intervention.

**Supplementary Information:**

The online version contains supplementary material available at 10.1186/s12943-021-01480-x.

## Background

Prostate cancer (PCa) is a commonly diagnosed cancer in men and the leading cause of cancer-related death in western countries [1]. Localized prostatic cancer can be cured by radical prostatectomy. However, once a tumor migrates outside of the gland, incurable metastatic disease is inevitable. Metastasis of PCa is the main cause of death, which greatly reduces the lifespan and quality of life of patients [2, 3]. The current treatment option is limited and ineffective for patients with advanced stage metastatic PCa [4–6]. Therefore, identifying novel biomarkers and therapeutic targets for metastatic PCa is a clinical imperative.

Circular RNAs (circRNAs) are single-stranded, covalently closed RNA molecules, formed by back-splicing of pre-mRNAs, and originally considered to be a by-product of splicing errors [7]. However, many studies have revealed that circRNAs are essential regulators of gene expression and implicate in multiple biological processes of tumorigenesis via acting as microRNA (miRNA) sponges, RNA binding protein (RBP)-binding molecules, transcriptional regulators, or templates for protein translations [8]. As reported, circRNA has multiple functions in regulating cell development, differentiation, and metabolism. In particular, some studies claim that the abnormal expression of circRNA is related to the invasion, migration and metastasis of cancer cells in distant organs. For example, circCSNK1G3 promotes PCa cell growth by interacting with miR-181 [9]. CircNOLC1 promotes PCa progression by sponging miR-647 [10]. CircRHOBTB3 inhibits gastric cancer growth via miR-654-3p [11]. CircACTN4 boosts the progression of breast cancer by complexing with FUBP1 [12]. CircSPARC enhances the progression of colorectal cancer by recruiting FUS [13]. These tremendously large number of reports indicated that circRNA may act as an ideal biological target for diagnosis and treatment, including PCa. However, multiple of circRNAs and their specific the biological roles in the metastasis of PCa stills worth to be explored.

In our study, we found a circRNA, which is derived from exons 4 and 5 of the ZNF652 (circBase ID: hsa_circ_0003258), as a novel oncogene in PCa. We found that hsa_circ_0003258 was significantly up-regulated in PCa tissues, and associated with the metastasis of PCa cells. Mechanistically, hsa_circ_0003258 directly interacted with the RNA-binding protein IGF2BP3, and enhanced the stability of histone deacetylase 4 (HDAC4) mRNA, consequently resulting in the aggressive nature of PCa. Moreover, hsa_circ_0003258 may upregulate the expression of Rho GTPase activating protein 5 (ARHGAP5) to promote tumor progression by sponging miR-653-5p. This work indicates the essential roles of hsa_circ_0003258 in PCa progression.

## Materials and methods

### Patients and samples

Plasma and tissue samples were obtained from 18 PCa patients of the Nanfang Hospital (Guangzhou, China). Inclusion criteria: 1. Patients who were diagnosed as PCa by pathological diagnosis before operation; 2. Patients signed an informed consent form. Exclusion criteria: 1. Patients with second primary tumors, HIV or syphilis virus, severe liver, kidney or other systemic diseases, other malignant diseases; 2. Patients who received preoperative chemotherapy or radiotherapy before surgery.

Nine healthy individuals who were in good health determined by physical examinations in the Nanfang Hospital were included as controls. The research protocol and the use of human tissues and plasma samples were approved by the Ethics Committee of Southern Medical University and all participants signed informed consents. A PCa tissue microarray (TMA) was purchased from Shanghai Outdo Biotech Co. Ltd. and contained both normal prostate tissues and PCa tissues along with each patient’s age, clinical stage, Gleason score, and metastasis status and tumor node metastasis (TNM) classification, that were recorded and archived in the National Engineering Center for Biochip. The detailed clinic features of enrolled patients were summarized in Supplementary Table S2. The TMA consisted of 134 cancer cases and were used for staining of has_circ_0003258.

### CircRNA Array analysis

The circRNAs from the plasm of PCa patients and control individuals were collected for microarray analysis. Methods of extracting plasma RNA was based on the previous protocols [14]. Sample preparation was performed according to the Arraystar’s standard protocols, as described previously [15]. Briefly, Using Rnase R to digest linear RNA and retain circRNAs. Then, RNAs were amplified for complementary RNA and labeled with an Arraystar Super RNA Labeling Kit (Kangcheng Biotechnology, Shanghai, China). Finally, these labeled RNAs were hybridized using Arraystar mouse circRNA Array (V1.0; Arraystar), and scanned by the Agilent Scanner G2505C (ANDbio, Temecula, CA). We defined the statistical criteria for selecting differentially expressed circRNAs using |fold changes| ≥ 2.0 with *p* values < 0.05.

### Quantitative real-time polymerase chain reaction (qRT-PCR) and genomic DNA extraction

TRIzol reagent (Invitrogen) was used to extract RNA from PCa cells and tissues. TB-Green PCR Master Mix Kit (Takara) and PrimeScript RT reagent Kit (TaKaRa) were used in qRT-PCR. Data were normalized to Glyceraldehyde 3-phosphate dehydrogenase (GAPDH) and used the 2^-ΔΔCt^ method to calculate. Genomic DNA (gDNA) is extracted from cells by the Easy Pure Genomic DNA kit (Transgen Biotech, Lot#L61221). All primers were obtained from Sangon Biotech and listed in Supplementary Table S1.

### Nuclear-cytoplasmic fractionation

Nuclear and cytoplasm of cells were separated by Nuclear and Cytoplasmic Extraction Reagents (Thermo Fisher Scientific, United States) Following the manufacturer’s instructions. Finally, Data were normalized to GAPDH (cytoplasmic control) and U6 (nuclear control) and used the 2^-ΔΔCt^ method to calculate.

### RNase R and Actinomycin D treatment

Total RNA (2 μg) was incubated with or without 3 U/mg of RNase R (Epicentre Technologies, Madison, WI, USA) for 15 min at 37 °C. The expression of hsa_circ_0003258 and other RNA were detected by qRT-PCR. PCa cells were treated with Actinomycin D or DMSO (Sigma Aldrich, St. Louis, MO, USA) to evaluate the stability of hsa_circ_0003258 and its linear gene ZNF652. The stability of RNA was detected by qRT-PCR.

### Fluorescence in situ hybridization (FISH)

The location of hsa_circ_0003258 was detected by FISH assay using a Cy3-labeled probes.

FISH kit (RiboBio, China) was used to examine the signals following the manufacturer’s instruction. The Nikon AISi Laser Scanning Confocal Microscope (Nikon instruments Inc., Japan) was used to visualize the images.

### Cell culture and transfection

PCa cell lines (LNCaP, C4–2, 22Rv1, PC3 and DU145) and human epidermal cell (RWPE-1) were purchased from the Stem Cell Bank, the Chinese Academy of Sciences. PCa cells lines were grown with RPMI-1640 medium (Gibco BRL, Grand Island, NY, USA) supplemented with 10% fetal bovine serum (Gibco, Australia). RWPE-1 cells were cultured in Keratinocyte Serum Free Medium (KSFM, Gibco). Cells were incubated at 37 °C in 5% CO_2_. Use Lipofectamine 3000 (Invitrogen, Carlsbad, CA) to transfect cells with designated nucleotides or plasmids according to the manufacturer’s instructions. Small interfering RNAs (siRNAs) targeting hsa_circ_0003258, IGF2BP3, HDAC4, ARHGAP5 and negative control (NC) siRNA were provided by RiboBio Co. (Guangzhou, China). All siRNA sequences were listed in Supplementary Table S1.

### RNA sequence and data analyses

RNA-seq (RNA sequencing) between the control DU145-NC and sh-has_circ_0003258 cell was performed as previously described [3]. RNA-seq is performed by BGISEQ platform. The other analyses including heatmap, gene set enrichment analysis was completed by BGI Dr. Tom system.

### Western blotting

Western blot analysis was performed as previously described [3]. In brief, PCa cells were added in radio immunoprecipitation assay buffer (RIPA, KeyGEN, BioTECH, China), and BCA Protein Assay Kit (KeyGEN, BioTECH, China) was used for protein determination. Then, the proteins were separated by 10% SDS-PAGE and transferred to polyvinylidene fluoride (PVDF) membranes (Millipore, USA). The membranes were blocked in no fat milk and then incubated with primary antibodies overnight at 4 °C. The primary antibodies included anti-Tubulin antibody (Cell Signaling Technology, USA, 1:1000), anti-ARHGAP5 antibody (ABclonal, China, 1:1000), anti-HDAC4 antibody (ABclonal, China, 1:1000), anti-IGF2BP3 antibody (Abcam, USA, 1:1000), anti-ZEB-1 antibody (Cell Signaling Technology, USA, 1:1000), anti-E-Cadherin antibody (Cell Signaling Technology, USA, 1:1000), anti-Vimentin antibody (Cell Signaling Technology, USA, 1:1000), and phospho-Erk1/2-T202/Y204 antibody (ABclonal, China, 1:1000). Subsequently, the membranes were immersed with the secondary antibody for 1 h. Finally, enhanced chemiluminescence (ECL) kit (Pierce Biotechnology, USA) was used to detect the level of protein.

### Transwell migration assay

Transwell (Costar, Corning, USA) with a multipolar (8.0 μm) polycarbonate membrane was utilized to conducted Cell migration experiments. Cells (5 × 10^4^) were mixed with serum-free medium and added into the upper chamber of the insert. Then, 800 μl complete medium was added to the bottom chamber. The cells in the chamber were mixed in 4% paraformaldehyde for 10 min and stained with Giemsa (Boster Ltd., Wuhan, China) at different time points. After that, cells in the upper chamber were removed and the number of cells on the bottom surface were observed under a microscope and counted using Image J software.

### Wound-healing assay

PCa cells were grown to confluence in six-well plates before making a wound. Using a 10 μl pipette tip to draw a gap and keep the cells in RPMI-1640 medium for 24 h. The images of the wound area were taken using the microscope at 0, 12 and 24 h. Wound widths were measured using the ImageJ software.

### Immunofluorescence

PCa cells were grown on coverslips (Corning, USA), and mixed with 4% paraformaldehyde for 30 min. Then the cells were permeabilized with 0.1% TritonX-100. Subsequently, the cells were incubated with Tris-buffered saline containing 5% bovine serum albumin (BSA) for 30 min. Afterwards, samples were incubated with antibodies specific for IGF2BP3 (Abcam, USA, 1:100) at 4 °C overnight. Finally, coverslips were treated with the fluorescent secondary antibody Alexa Fluor 488-conjugated goat anti-rabbit IgG (#4412S, Cell Signaling Technology) (1250 dilution) and DAPI (300 nmol/L) staining. The images were photographed under a Nikon AISi Laser Scanning Confocal Microscope (Nikon instruments Inc., Japan).

### Luciferase reporter assay

Dual-Luciferase Reporter Assay kit (Genecopoaie, China) was used to verify the relationships between hsa_circ_0003258 and miR-653-5p. Wild-type (WT) or mutant (MUT) 3′-UTR of hsa_circ_0003258 were cloned into the firefly-tagged pGL3 promoter luciferase vector (GeneCopoeia, Rockville, USA). Using Lipofectamine 3000 (Invitrogen) to transfect HEK-293 cells with miR-653-5p mimic hsa_circ_0003258 or its mutant plasmid following the manufacturer’s instructions. Luciferase activities were detected by a dual luciferase assay system (GeneCopoeia) after 48 h. The experiment was performed with three replicates.

### Biotinylated RNA pull-down assay

Biotin-labeled hsa_circ_0003258 and oligonucleotide probes (RiboBio Co. Ltd. Guangzhou, China) were mixed with streptavidin magnetic beads (Beaver, China) in RIP buffer for 4 h. Subsequently, the DU145 cell lysate was incubated with the probes complex for 12 h at 4 °C. After purification, enriched hsa_circ_0003258 and miRNAs were quantified by qRT-PCR. Meanwhile, the bound proteins were identified by Western blotting.

### RNA immunoprecipitation assay

RIP assay was performed by using EZ-Magna RIP™ Kit (#17–701, Merck Millipore) with antibodies specific for IGF2BP3 following the manufacturer’s instructions. The immunoprecipitated RNAs were detected by qRT-PCR to measure the level of hsa_circ_0003258 and HDAC4. Total RNAs (input) and isotype antibody (IgG) were applied as controls.

### Animal models

All experiments’ animal procedures were approved by the Animal Care Committee of Southern Medical University. For tumor metastatic studies in vivo, DU145 cells (1 × 10^7^ cells per mouse) transfected with NC or sh-has_circ_0003258 were injected through lateral tail vein of BALB/c nude male mice (*n* = 5 for each group). Lung tissues were collected and examined for metastasis. After 40 days, the tumors in vivo were evaluated by fluorescence imaging using the IVIS (PerkinElmer, USA). The presence of cancer cells was confirmed by H&E (hematoxylin and eosin) staining. At the same time, immunohistochemistry (IHC) staining was conducted using antibodies against ARHGAP5 (Abcam, USA, 1:100) and HDAC4 (Abcam, USA, 1:100).

### Statistical analyses

Statistical analyses were performed using the SPSS software version 20.0 (SPSS, Inc., Chicago, IL) or GraphPad Prism 7.0 (GraphPad Software, USA). All values are expressed as mean ± standard deviation (SD). Differences in mean values between groups were analyzed using ANOVA and Student’s t tests. The correlation between hsa_circ_0003258 expression and clinicopathological properties was analyzed using a χ^2^ test. *P* value < 0.05 was considered statistically significant. (*, *P* <0.05. **, *P* ≤ 0.01. ***, *P* ≤ 0.001. ****, *P* ≤ 0.0001).

## Results

### Has_circ_0003258 is up-regulated in PCa tissues and correlated with aggressive progression of PCa

**Fig. 1:**
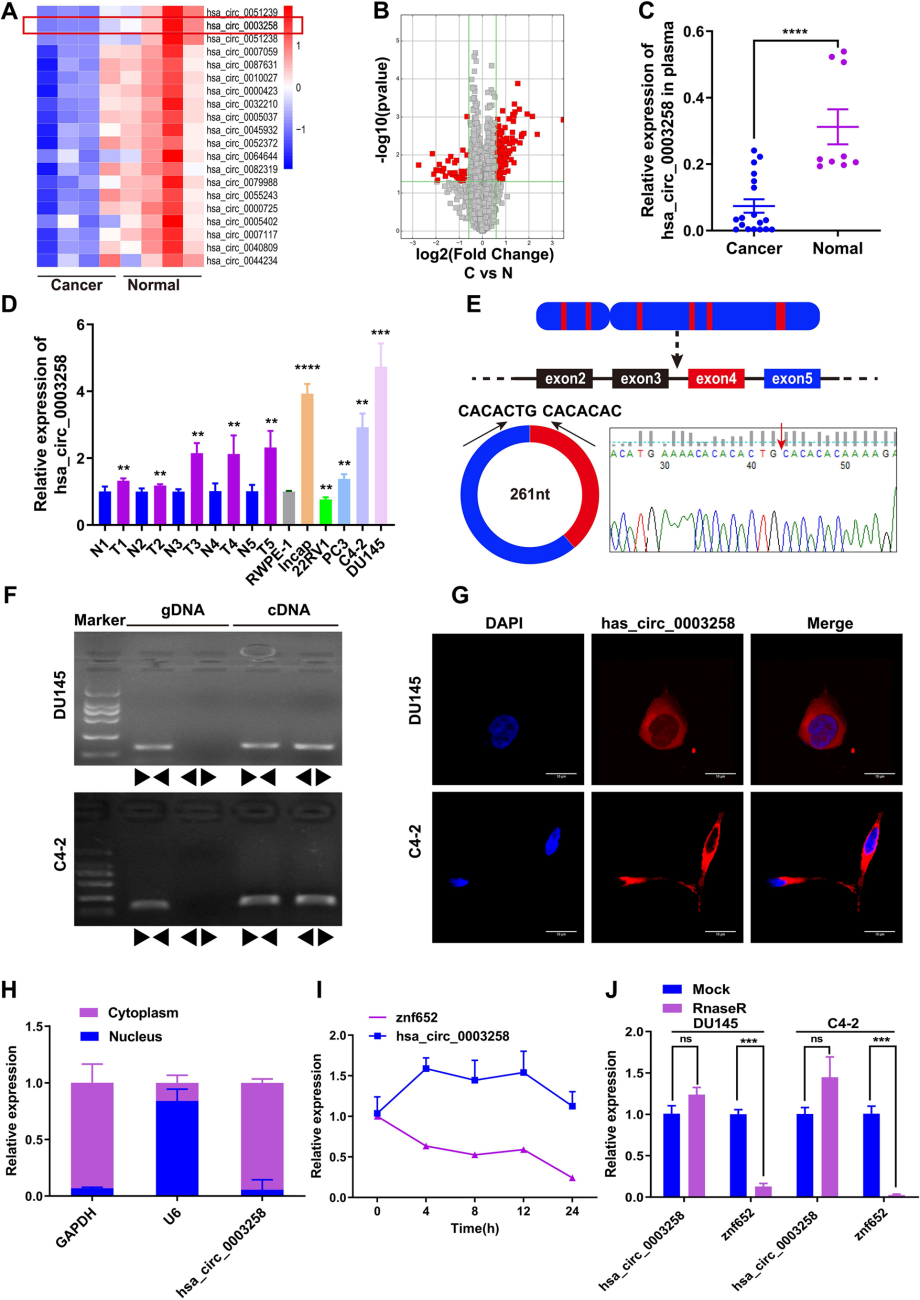
Screening and characterizing hsa_circ_0003258 in PCa. **A** The heat map showing the top 20 down-regulated circRNAs in prostate cancer plasma samples compared with non-tumor plasma samples analyzed by RNA sequencing. **B** The volcano plot showing the expression profile of circRNAs in prostate cancer plasma samples. **C** hsa_circ_0003258 expression evaluated by qRT-PCR in plasma samples of prostate cancer patients. **D** qRT-PCR for the expression of hsa_circ_0003258 in PCa cell lines and tissues. The expression of hsa_circ_0003258 was normalized to GADPH. **E** Schematic illustration indicating the generation of hsa_circ_0003258 from its host gene, and validation by Sanger sequencing. **F** PCR assay with divergent and convergent primers showing the amplification of circRNAs from cDNA or genomic DNA (gDNA) of prostate cancer cell lines. **G** RNA FISH detecting hsa_circ_0003258’s subcellular localization in DU145 and C4–2 cells. Nuclei was stained with DAPI. Scale bar, 10 μm. **H** QRT-PCR value indicating the abundance of hsa_circ_0003258, U6 and GAPDH in either the cytoplasm or nucleus of DU145 and C4–2 cells. Hsa_circ_0003258 was normalized to GAPDH in the cytoplasm and U6 in the nucleus **I** QRT-PCR for abundance of hsa_circ_0003258 and ZNF652 mRNA in DU145 cells treated with Actinomycin D at indicated time point. **J** The relative hsa_circ_0003258 or linear ZNF652 mRNA abundance detected by qRT-PCR after treated with or without RNase R in DU145 and C4–2 cells. ns: Not Significant; ** *P* < 0.01; *** *P* < 0.001; **** *P* < 0.0001

In order to investigate the role of circRNA in the development of PCa, four pairs of plasma samples (from 4 patients and 4 normal individuals) were used for circRNA microarray analysis to examine the expression of circRNAs in these samples. The result showed that 7131 circRNAs were found in the PCa group and normal group (Fig. S1A). The top 20 down-regulated circRNAs were listed in Fig. 1A. The variation of circRNA expression between cancer and normal samples were showed in volcano plots (Fig. 1B). QRT-PCR was performed to verify the microarray results. The results showed that it was consistent with the microarray results. Compared with normal samples, the level of has_circ_0003258 in PCa plasma was down-regulated (Fig. 1C).

To verify the circRNA sequencing results, we detected the expression of has_circ_0003258 in PCa cells and tissues (Fig. 1D). We found Has_circ_0003258 was significant up-regulated in PCa cells. Next, we validated the expression of has_circ_0003258 in PCa tissues and normal prostate tissues through FISH assays (Fig. S1B). Clinicopathological features showed that up-regulation of has_circ_0003258 was positively associated with TNM stage and Grade (Table 1). The level of has_circ_0003258 was not associated with the age of patients.

**Table 1:** Correlation between hsa_circ_0003258 expression and clinicopathologic characteristics in PCa patients

Variables	n	hsa_circ_0003258 expression		***P*** value^**c**^
		Low (%)	High (%)	
**Type**				**<0.001**
BPH^a^	43	40 (93)	3 (7)	
PCa^b^	91	12 (13.2)	79 (86.8)	
**Age**				0.904
≤70	39	20 (51.3)	19 (48.7)	
>70	52	26 (50.0)	26 (50.0)	
**Grade**				**<0.001**
≤3	48	34 (70.8)	14 (29.2)	
>3	43	12 (27.9)	31 (72.1)	
**T stage**				**<0.001**
<3	15	13 (86.7)	2 (13.3)	
≥3	76	33 (43.4)	43 (56.6)	
**N stage**				**<0.001**
N0	71	43 (60.6)	28 (39.4)	
N1	20	3 (15.0)	17 (85.0)	

^a^benign prostatic hyperplasia

^b^*PCa* prostate cancer

^c^*p* value is from χ^2^-test -test

### Characterization of hsa_circ_0003258 in PCa

According to the human reference genome (GRCh37/hg19) acquired from the UCSC genome database (http://genome.ucsc.edu/), we found that the genomic length of the hsa_circ_0003258 is 731 bp and the spliced length is 261 bp. We identified that has_circ_0003258 is derived from ZNF652, which is located on chromosome 17q21 from CircBase (http://www.circbase.org/). Then, we examined the structure of hsa_circ_0003258. Hsa_circ_0003258 was formed by the back-splicing of exon 4 and 5 of linear gene ZNF652 (Fig. 1E). PCR with an electrophoresis assay showed that hsa_circ_0003258 could be amplified by outward-facing divergent primers in cDNA. Additionally, hsa_circ_0003258 could not be amplified from gDNA and the production was confirmed by Sanger sequencing (Fig. 1E-F). These data demonstrated that hsa_circ_0003258 contained two circularized exons, formed from exon back-splicing. FISH analysis (Fig. 1G) and mRNA fractionation (Fig. 1H) revealed that the majority of hsa_circ_0003258 preferentially localized in the cytoplasm. Analysis of coding potential showed that hsa_circ_0003258 lacks protein coding ability (Fig. S1C). The melting curve of hsa_circ_0003258 amplified product using divergent primers showed a single peak the same as GAPDH (Fig. S1D). Notably, due to the high stability of the circular isoform, the half-life of the transcript exceeds 24 h (Fig. 1I) in DU145 and C4–2 cells. In addition, resistance to RNase R confirms that RNA species was circular in form (Fig. 1J). We found that hsa_circ_0003258 was more stable than ZNF652 owing to its circular structure. These results demonstrated that hsa_circ_0003258 is a stable circRNA expressed in PCa cells.

### Hsa_circ_0003258 promotes the metastasis of PCa in vitro

**Fig. 2:**
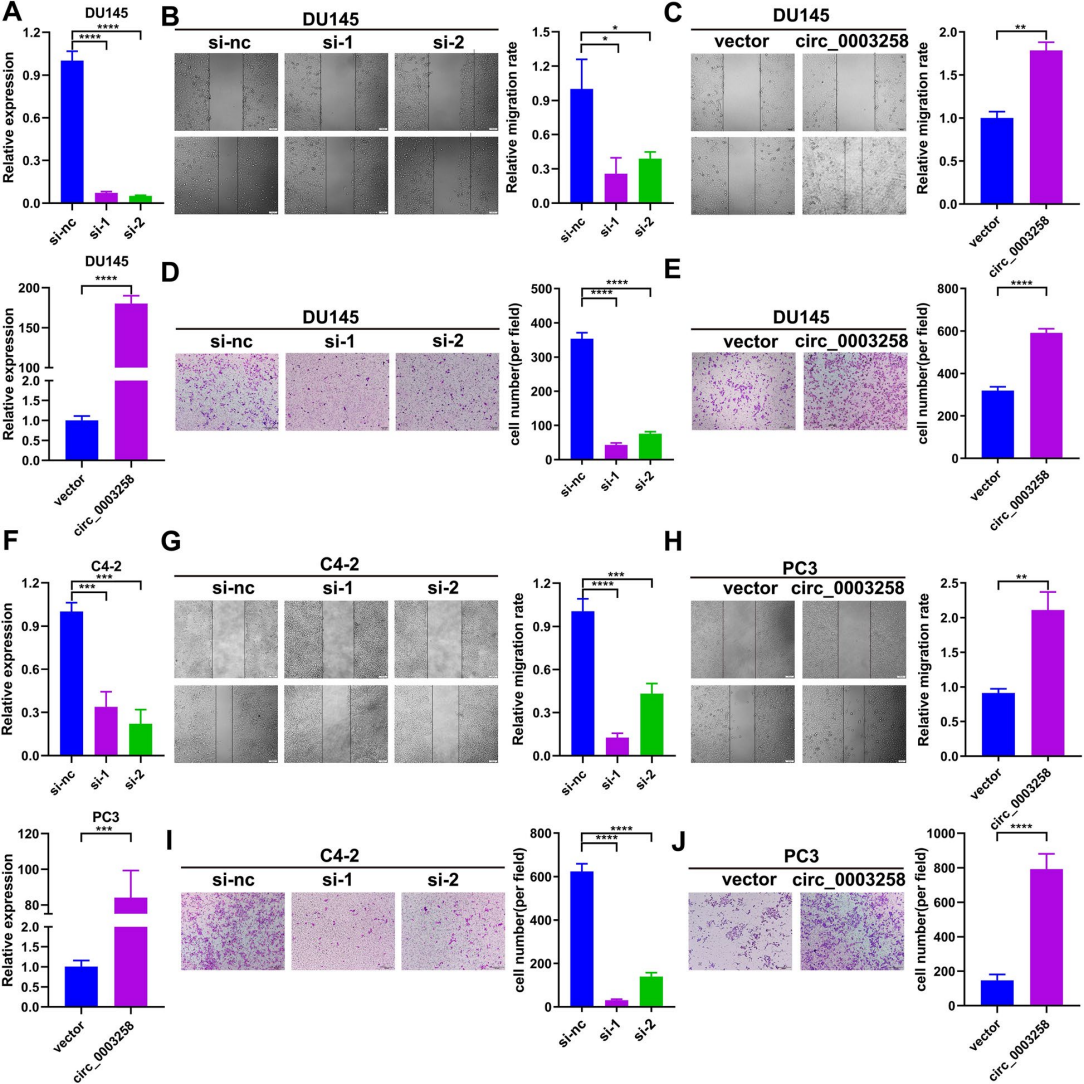
Hsa_circ_0003258 promotes the aggressiveness of PCa cells. **A** DU145 cells transfected with two siRNAs specifically targeting hsa_circ_0003258(S1, S2) and hsa_circ_0003258 overexpression plasmid for 48 h, and the level of hsa_circ_0003258 was detected by qRT-PCR. **B**-**C** Wound healing assay detected the migratory capacity of PCa cells after changing the level of hsa_circ_0003258. Scale bar, 100um. **D**-**E** Transwell assay detected the migratory capacities of PCa cells after changing the level of hsa_circ_0003258. Scale bar, 100um. **F** C4–2 cells transfected with two siRNAs specifically targeting hsa_circ_0003258(S1, S2) and PC3 cells transfected with hsa_circ_0003258 overexpression plasmid for 48 h, and the level of hsa_circ_0003258 detected by qRT-PCR. **G**-**H** Wound healing assay detected the migratory capacity of PCa cells after changing the level of hsa_circ_0003258. Scale bar, 100um. **I**-**J** Transwell assay detected the migratory capacity of PCa cells after changing the level of hsa_circ_0003258. Scale bar, 100um. * *P* < 0.05; ** *P* < 0.01; *** *P* < 0.001; **** *P* < 0.0001

To evaluate the functions of hsa_circ_0003258 in PCa, we used siRNA which specifically targeted the back-splicing junction of hsa_circ_0003258. RT-PCR analyses revealed that si-hsa_circ_0003258 significantly silenced the intracellular hsa_circ_0003258, while the overexpression group showed increased level of hsa_circ_0003258 compared with the control (Fig. 2A and F). Then we evaluated the effects of hsa_circ_0003258 on PCa cell migration capacity by wound healing and transwell migration assays. We found that downregulation of hsa_circ_0003258 significantly decreased the migratory capabilities of PCa cells. However, wound healing and transwell assays showed that overexpression of hsa_circ_0003258 remarkably enhanced wound healing and migratory capabilities of PCa cells (Fig. 2B-E and 2G-J). These results revealed that the migration of various PCa cells were not impaired by the negative siRNAs or vector plasmid (Fig. S1E-H), suggesting the importance of hsa_circ_0003258 in promoting the aggressiveness of PCa cells. Besides, the CCK-8 and colony formation assays revealed that hsa_circ_0003258 depletion had no effect on cell proliferation (Fig. S1I-J).

### Hsa_circ_0003258 promotes the metastasis of PCa via stimulating EMT through activating ERK signaling pathway

**Fig. 3:**
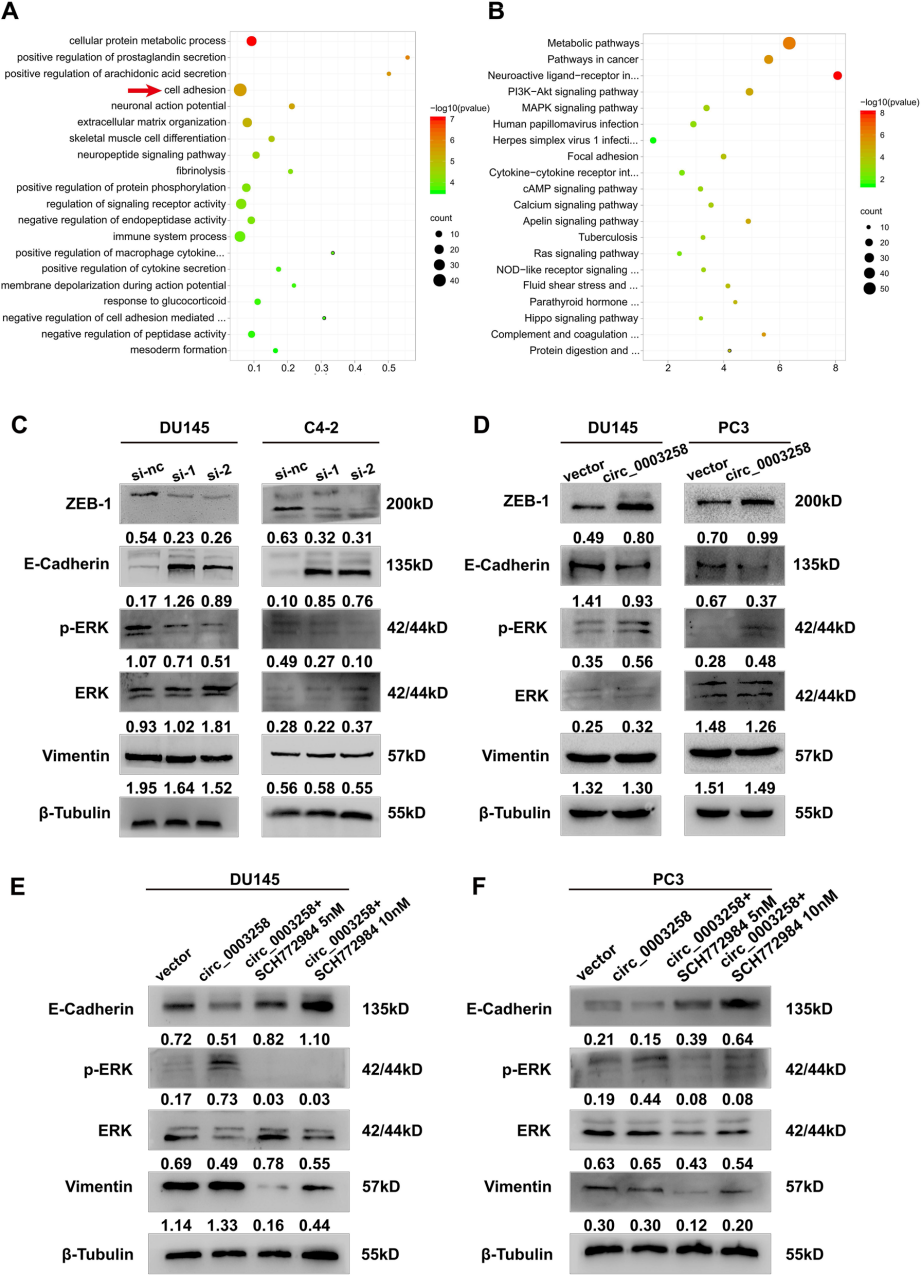
Hsa_circ_0003258 promotes the EMT of prostate cancer through MAPK signaling pathway in vitro. **A-B** The enriched GO and KEGG functional pathways. **C**-**D** Western blot showing the protein level in cells with overexpression or silencing of hsa_circ_0003258. **E**-**F** Western blot analysis evaluated the migratory capacity and expression of p-ERK, ERK and EMT biomarkers in DU145- and C4–2- vector/Lv- hsa_circ_0003258 cells treated with SCH772984 for 48 h

To explore the mechanisms underlying the effects of hsa_circ_0003258 on PCa cell metastasis, we screened the signaling pathways that might be involved in hsa_circ_0003258 by using RNA-seq analysis in DU145 cells. We constructed stable knockdown DU145 cells using sh-hsa_circ_0003258 lentiviral system. Compared with the control cells, 166 genes were upregulated and 532 genes were downregulated in the sh-hsa_circ_0003258 cells (Fig. S2A). Gene Ontology (GO) analysis showed that differential mRNAs are enriched in cell adhesion (Fig. 3A). KEGG pathway enrichment analysis indicated that the differentially expressed genes induced by hsa_circ_0003258 knockdown were enriched in the MAPK signaling pathway (Fig. 3B). EMT enables epithelial cells to acquire migratory and invasive capabilities, so we investigated whether hsa_circ_0003258 affects migration of PCa cells by regulating the expression of EMT related proteins. The expression of E-cadherin, Vimentin and ZEB-1 were examined in hsa_circ_0003258-depleted or overexpressed PCa cells (Fig. 3C-D). Given the crucial role of the MAPK signaling pathway in the development of PCa, we investigated whether hsa_circ_0003258 could modulate the MAPK signaling pathway. Western blotting showed that the p-ERK levels were markedly decreased following knockdown of hsa_circ_0003258 in DU145 and C4–2 cells. The opposite effect was observed following the overexpression of hsa_circ_0003258 (Fig. 3C-D). In addition, treatment with ERK inhibitor SCH772984 partially restored EMT expression and decreased p-ERK in DU145 and C4–2 cells (Fig. 3E-F). Transwell assays demonstrated that inhibition of ERK signaling blocked the PCa cell migration (Fig. S2B). These results suggest hsa_circ_0003258 is a positive regulator of the ERK signaling pathway.

### Hsa_circ_0003258 function as a sponge for miR-653-5p

**Fig. 4:**
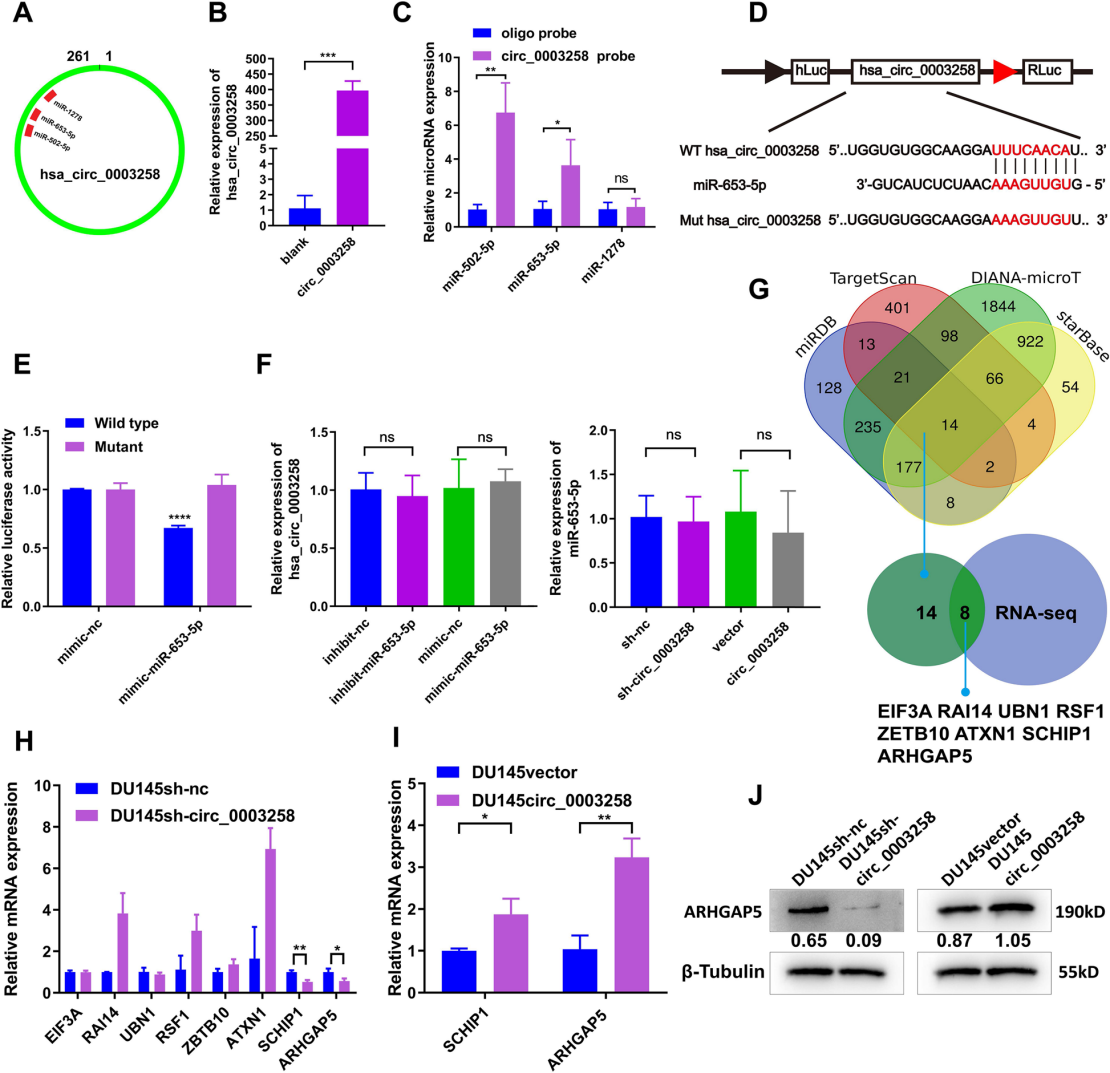
Hsa_circ_0003258 serves as a sponge for miR-653-5p in PC cells. **A** Schematic drawing showing the putative binding sites of miRNAs related to hsa_circ_0003258. **B** Lysates from DU145 cells subjected to biotinylated-hsa_circ_0003258 pull-down assay and the expression levels of hsa_circ_0003258 measured by qRT-PCR. **C** The expression of the top three candidate miRNAs predicted by CircInteractome database were quantified by qRT-PCR after biotinylated-hsa_circ_00032583 pull-down assay in DU145 cells. **D** A schema of hsa_circ_0003258 wild-type (WT) and mutant (Mut) luciferase reporter vectors. **E** Luciferase activity was tested in DU145 cells co-transfected with luciferase reporter containing hsa_circ_0003258 sequences with wild type and mutant binding site of miR-653-5p and the mimic of miR-653-5p or control. **F** left The relative expression of miR-653-5p detected by qRT-PCR after over expression or silencing of hsa_circ_0003258 in DU145 cells. **F** right The relative expression of hsa_circ_0003258 detected by qRT-PCR after the transfection of miR-653-5p mimic or inhibitor. **G** Venn diagram showing the predicted target genes of miR-653-5p. **H** The qRT-PCR showing the mRNA level change of the predicated targets after silencing of hsa_circ_0003258. **I** The qRT-PCR showing the mRNA level change of the predicated targets after over expression of hsa_circ_0003258. **J** Western blot showing protein levels of ARHGAP5 after overexpression or silencing of hsa_circ_0003258. ns: Not Significant; * *P* < 0.05; ** *P* < 0.01; *** *P* < 0.001; **** *P* < 0.0001

CircRNAs have been reported to act as miRNA sponges. We assessed whether hsa_circ_0003258 could sponge miRNAs to affect PCa progression. To investigate whether hsa_circ_0003258 could sponge miRNA in PCa cells, we selected the 3 top potential miRNAs (miR-1278, miR-502-5p, miR-653-5p) with a score ≥ 90 predicted by the CircInteractome database (Fig. 4A and S2C). We designed a 3′ terminal-biotinylated-hsa_circ_0003258 probe to explore which miRNAs combined with hsa_circ_0003258. As shown in Fig. 4B and S2D, the probe was used to pull down hsa_circ_0003258 in DU145 cell line. By RIP hsa_circ_0003258 pull down experiments, we purified the hsa_circ_0003258-associated RNAs and analyzed the 3 candidate miRNAs in the complex. We found a specific enrichment of hsa_circ_0003258 and miR-653-5p and miR-502-5p as compared to the controls, while the other miRNAs had no enrichment, suggesting that miR-653-5p and miR-502-5p are the hsa_circ_0003258-associated miRNA in PCa cells (Fig. 4C and S2E). Since the interaction of miR-502-5p and hsa_circ_0003258 had been previously reported, we investigated the interaction of miR-653-5p and hsa_circ_0003258. To further verify that miR-653-5p can bind to hsa_circ_0003258, HEK-293 T cells were performed to detect dual-luciferase activity. Wild type (WT) hsa_circ_0003258 and mutant type (MUT) hsa_circ_0003258 were cloned into the luciferase reporter vector pLG3. The results showed miR-653-5p mimic significantly reduced the luciferase activity of the WT-hsa_circ_0003258 (Fig. 4D-E). In addition, after silencing or overexpressing hsa_circ_0003258 in DU145 cells, the level of miR-653-5p did not show a significant change (Fig. 4F left), while the expression of hsa_circ_0003258 after transfection with miR-653-5p mimics or inhibitors Shows no significant changes (Fig. 4F right and S2F). These results indicated that hsa_circ_0003258 promote PCa progression via miR-653-5p.

### Hsa_circ_0003258 promotes PCa metastasis through hsa_circ_0003258/ miR-653-5p/Arhgap5 pathway

**Fig. 5:**
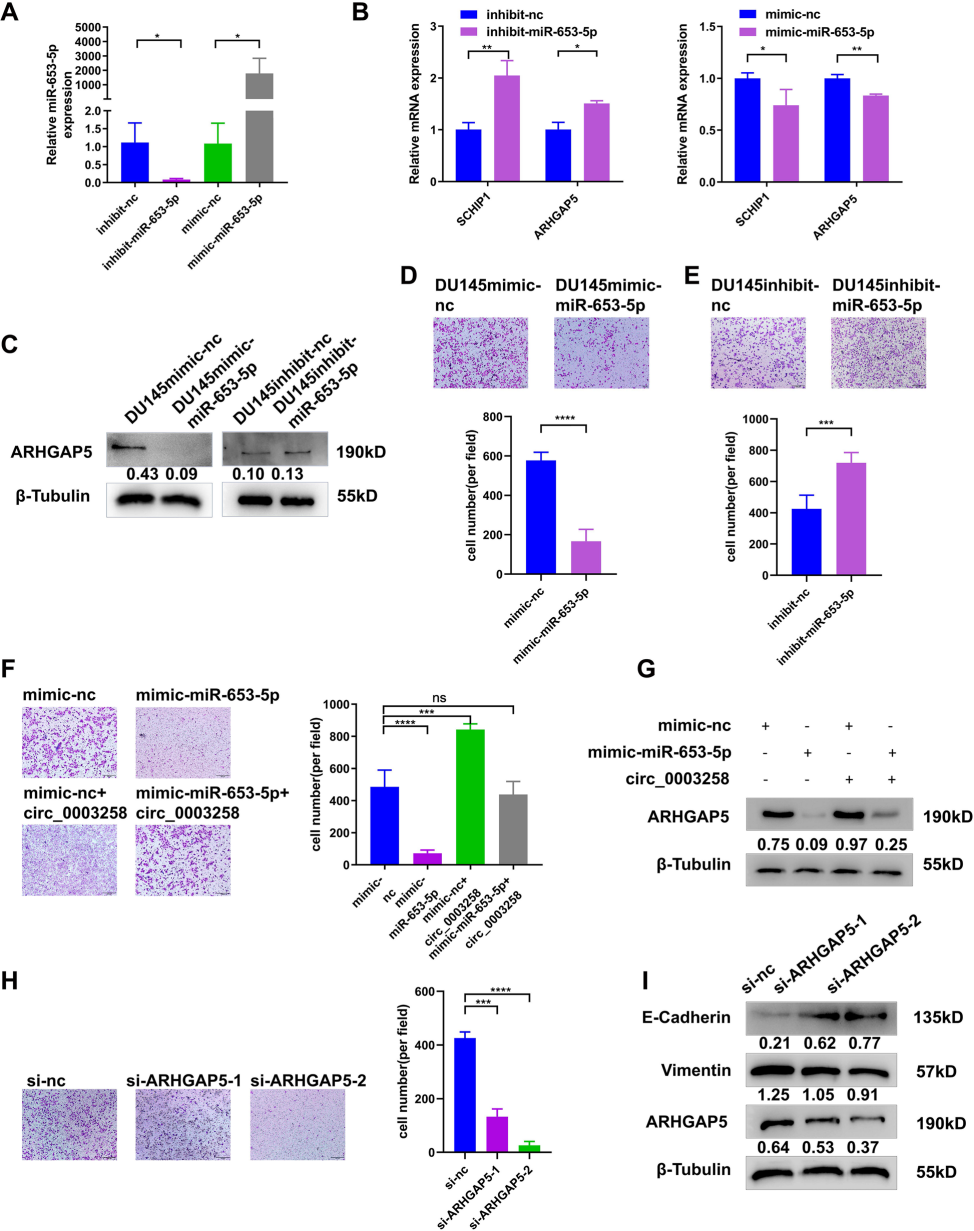
Hsa_circ_0003258 promotes PCa metastasis through the hsa_circ_0003258/ miR-653-5p/Arhgap5 pathway. **A** QRT-PCR assay explored the overexpression and knockdown efficiency of miR-653-5p. **B** The qRT-PCR showing the ARHGAP5 mRNA level of the predicated targets with miR-653-5p mimic or miR-653-5p inhibitor in PCa cells. **C** Western blot showing the protein levels of ARHGAP5 after overexpression or silencing of miR-653-5p. **D**-**E** Cell migration observed in transwell assay after overexpression or silencing of miR-653-5p. **F**-**G** Transwell assay and Western blot was performed after transfection with indicated vectors, lv- hsa_circ_0003258, mimic-nc or mimic-miR-653-5p. **H**-**I** Transwell assay and Western blot detected the migratory capacity and the protein level of PCa cells after silencing ARHGAP5. ns: Not Significant; * *P* < 0.05; ** *P* < 0.01; *** *P* < 0.001; ****P < 0.0001

According to the results, we further investigated the target genes of miR-653-5p that play critical roles in the metastasis of PCa. Firstly, we predicted the target in miRDB, DIANA-microT, Targetscan and Starbase. Secondly, we analyzed the differential gene expression in DU145 cells (hsa_circ_0003258-sh vs. hsa_circ_0003258-nc) by RNA-seq. According to the prediction of target genes and the results of RNA-seq, we found 8 differentially expressed genes (SCHIP1, UBN1, EIF3A, ZBTB10, ARHGAP5, ATXN1, RSF1, and RAI14) (Fig. 4G). The mRNA expression of candidate genes was detected after silencing hsa_circ_0003258 and found that ARHGAP5, SCHIP1 were significantly increased in DU145- and C4–2-Lv cells and decreased in DU145- and C4–2-sh cells (Fig. 4H-I and S2G). In addition, under the regulation of hsa_circ_0003258, the protein expression of ARHGAP5 altered with the same trend as the mRNA levels (Fig. 4J and S2H). Then the mRNA and protein expression of these targets (ARHGAP5, SCHIP1) were significantly decreased by miR-653-5p mimics and upregulated by miR-653-5p inhibitors in PCa cells (Fig. 5B-C and S2I-J). The expression of miR-653-5p is shown in Fig. 5A after transfection with the mimic and inhibitor. In addition, transwell assay showed that overexpression of miR-653-5p and knockdown of miR-653-5p expression decreases and increases PCa cell metastasis, respectively (Fig. 5D-E and S3A).

Based upon, we assumed that hsa_circ_0003258 accelerated the migration capacity of PCa by protecting ARHGAP5 from decrease of miR-653-5p. Firstly, the transwell assay showed that mimic-miR-653-5p in hsa_circ_0003258-overexpression cells impaired the migration of DU145 cells (Fig. 5F and S3B). Secondly, we found that hsa_circ_0003258 increased the expression of ARHGAP5 in DU145 cells, which was reversed by miR-653-5p mimics; while the silence of hsa_circ_0003258 inhibited the expression of ARHGAP5 in DU145 cells, and miR-653-5p inhibitors could reverse the expression (Fig. 5G and S3C). Thirdly, we determined whether ARHGAP5 promoted EMT of PCa cells. Our results showed that downregulation of ARHGAP5 significantly decreased the number of migratory PCa cells (Fig. 5H and S3D). Moreover, Western blot analysis showed that depletion of ARHGAP5 markedly changed the level of E-cadherin protein (Fig. 5I). These results indicated that hsa_circ_0003258 releases ARHGAP5 by adsorbing miR-653-5p, which promoted metastasis of PCa.

### The RNA-binding protein IGF2BP3 binds to hsa_circ_0003258 in PCa cells

**Fig. 6:**
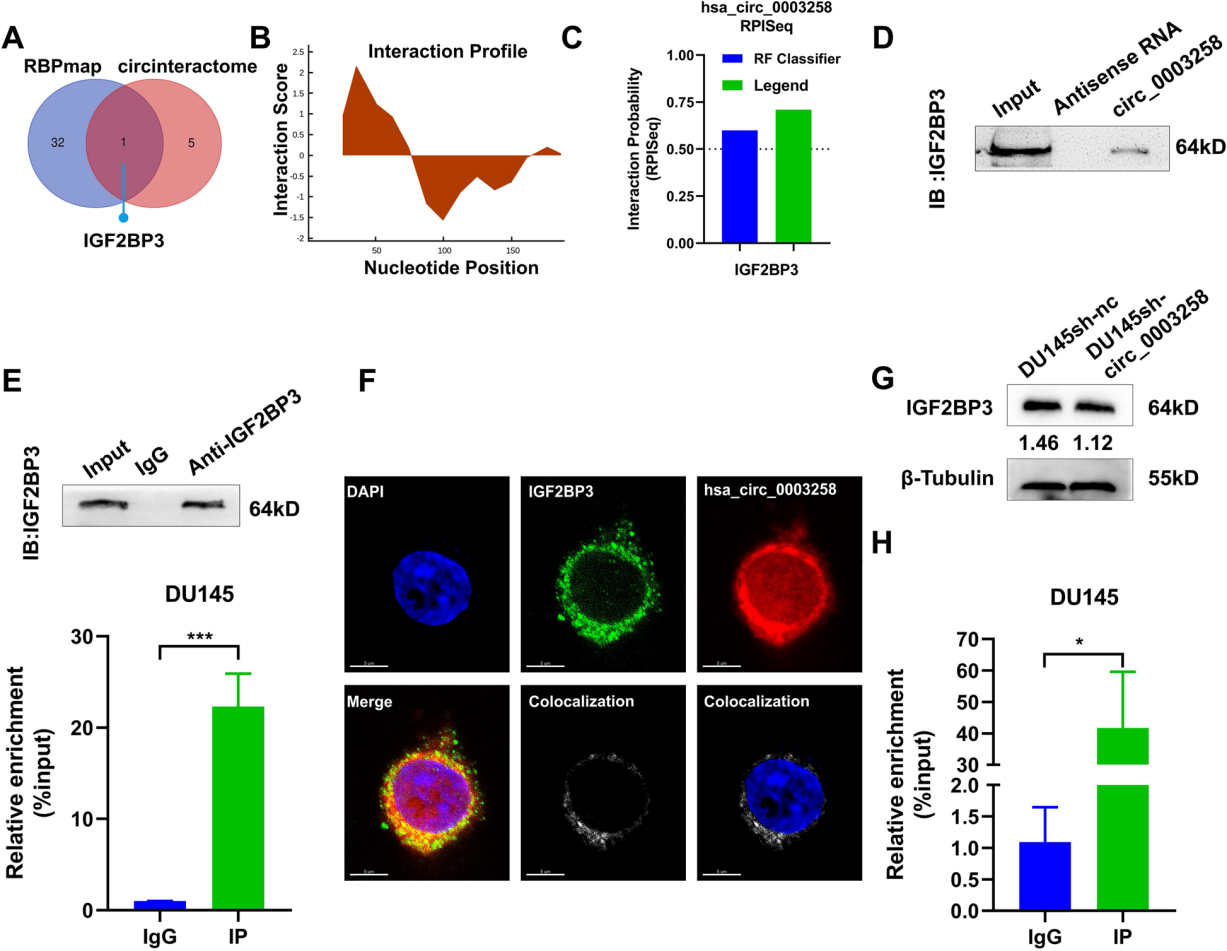
The RNA-binding protein IGF2BP3 binds to hsa_circ_0003258 in PCa cells. **A** Venn diagram showing the predicted RNA binding proteins. **B** The binding sites between hsa_circ_0003258 and IGF2BP3 were predicted online. **C** The interaction possibility of hsa_circ_0003258 and IGF2BP3 detected in RPISeq and the result showed that hsa_circ_0003258 bound to IGF2BP3. **D** Western blot analysis of IGF2BP3 levels in pulldown assays using a biotinylated antisense oligomer targeting the junction of hsa_circ_0003258. **E** RIP assays showing the association between IGF2BP3 and Hsa_circ_0003258. Top, IP efficiency of IGF2BP3-antibody shown in Western blotting. Bottom, relative enrichment representing RNA levels associated with IGF2BP3 relative to an input control. IgG antibody served as a control. **F** IF and FISH assay showing that hsa_circ_0003258 is colocalized with IGF2BP3 protein in the cytoplasm. **G** Western blot showing protein levels of IGF2BP3 after silencing of hsa_circ_0003258. **H** m6A bound to hsa_circ_0003258 performed by meRIP. * *P* < 0.05; *** *P* < 0.001

We explored the mechanisms of hsa_circ_0003258 in promoting PCa progression. Recent studies showed that circRNAs interact with a variety of proteins and participate in the molecular regulation of tumors. We identified hsa_circ_0003258-interacting proteins by CircInteractome (https://circinteractome.nia.nih.gov/) and RBPmap (http://rbpmap.technion.ac.il/) (Fig. 6A). Furthermore, we predicted the potential binding sites between hsa_circ_0003258 and IGF2BP3. Firstly, we analyzed the nucleotides sequence of hsa_circ_0003258 by catRAPID (http://s.tartaglialab.com/page/catrapid_group), and found that nucleotides at 0–80 had a high potential to bind with proteins (Fig. 6B). The possibility of the combination of the hsa_circ_0003258 and IGF2BP3 is shown in Fig. 6C. Based upon, IGF2BP3 was selected for our next study. Western blot analysis indicated the existence of IGF2BP3 within the hsa_circ_0003258 sense RNA probe pull-down samples in DU145 cells (Fig. 6D and S4A up). Meanwhile, RNA immunoprecipitation confirmed the interaction between IGF2BP3 and hsa_circ_0003258 in DU145 and C4–2 cells (Fig. 6E and S4A down). We confirmed the co-localization of hsa_circ_0003258 and IGF2BP3 in the cytoplasm through IF and FISH detection (Fig. 6F and S4B). These results indicated that hsa_circ_0003258 and IGF2BP3 form an RNA-protein complex in PCa cells. IGF2BP3 is overexpressed in PCa cells and correlates with high Gleason scores [16, 17]. Therefore, we explored the relationship between IGF2BP3 and hsa_circ_0003258. However, we observed that the overexpression of hsa_circ_0003258 did not affect the level of IGF2BP3 protein (Fig. 6G). Since IGF2BP3 is known as an m6A reader, we predicted the sites of m6A modification of hsa_circ_0003258 via SRAMP website tools. In the predicted result, hsa_circ_0003258 showed various m6A modification sites. Among them, there is a site with the highest confidence and another site with moderate confidence located in the hsa_circ_0003258 region (Fig. S4C). From methylated RNA immunoprecipitation (MeRIP) assay, hsa_circ_0003258 was enriched in the m6A precipitated fraction (Fig. 6H), confirming the m6A modification in hsa_circ_0003258.

### Hsa_circ_0003258 and IGF2BP3 complex promotes EMT in PCa cells through stabilizing HDAC4 mRNA

**Fig. 7:**
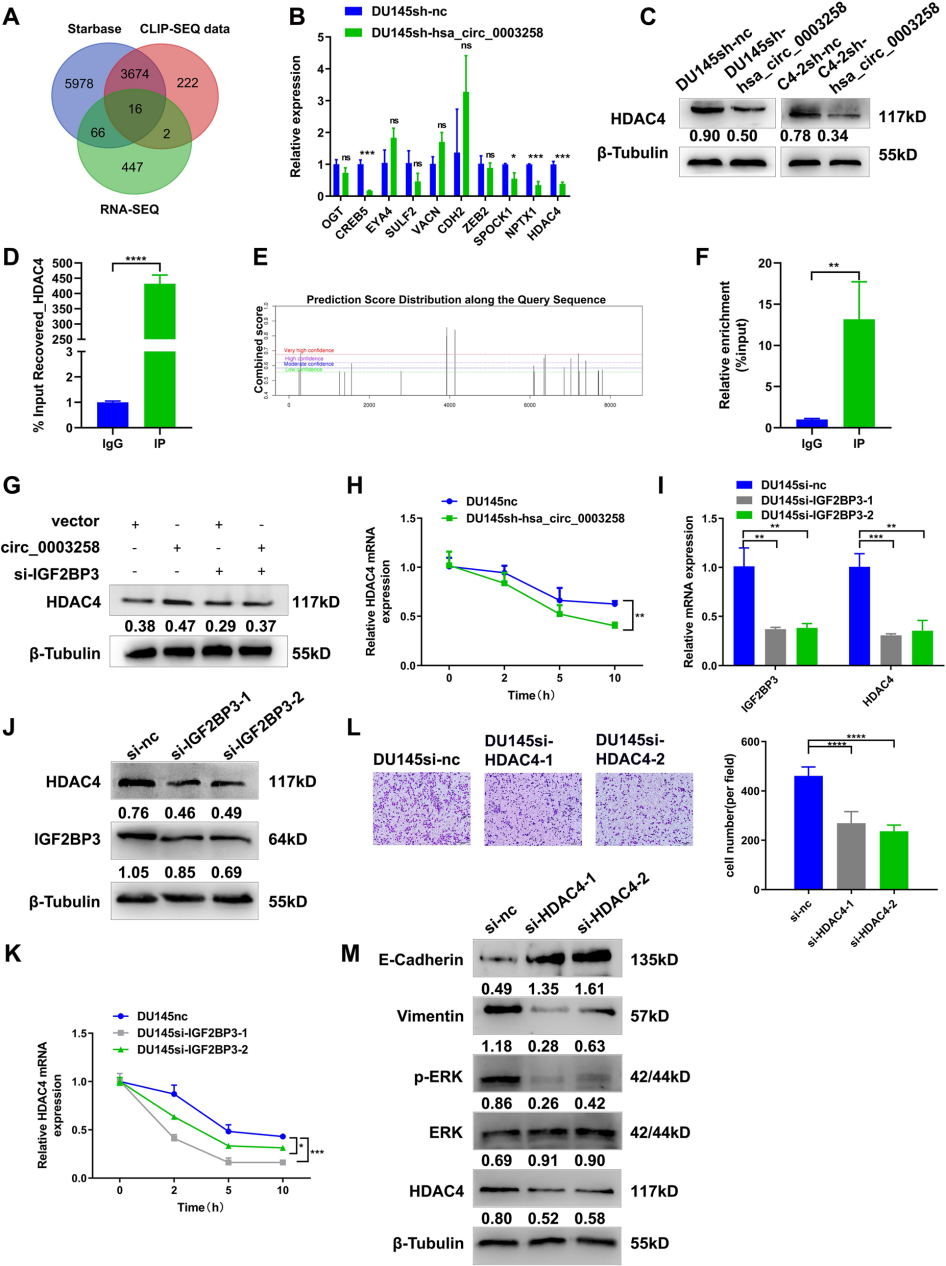
Hsa_circ_0003258 promotes PCa metastasis through HDAC4 pathway. **A** Venn diagram showing the RNA candidates as the targets of IGF2BP3. **B**. The qRT-PCR showing the mRNA level change of the predicated targets after silencing hsa_circ_0003258. **C** Western blot showing the protein level of HDAC4 after silencing hsa_circ_0003258. **D** RIP assays showing the association of IGF2BP3 with HDAC4. Relative enrichment representing RNA level associated with HDAC4 compared to an input control. IgG antibody served as a control. **E**-**F** The m6A modification site of HDAC4 predicted by SRAMP website tools and m6A bound to HDAC4 performed by meRIP. **G** HDAC4 protein expression determined by Western blot. **H** hsa_circ_0003258 depletion on the stability of HDAC4, cells were treated with Actinomycin D at indicated time points and mRNA levels of HDAC4 were validated with qRT-PCR. The U6 was used as normalization control. **I**-**J** QRT-PCR and Western blot assay examined the knockdown efficiency of IGF2BP3 and the protein level of HDAC4 after silencing of IGF2BP3. **K** IGF2BP3 depletion on the stability of HDAC4, cells were treated with Actinomycin D at indicated time points and mRNA level of HDAC4 were validated by qRT-PCR. The U6 was used as normalization control. **L**-**M** Transwell assay and Western blot were used to detect the migratory capacity and the protein level in PCa cells after silencing HDAC4. * *P* < 0.05; ** *P* < 0.01; *** *P* < 0.001; **** *P* < 0.0001

As IGF2BP3 is essential for mRNA stability, we investigated whether the hsa_circ_0003258/IGF2BP3 complex stabilizes downstream targets. RNA-seq analyses showed five hundred and thirty-two mRNAs were significantly downregulated in hsa_circ_0003258 silencing cells (fold change > 2.0). We screened the IGF2BP3-binding 3′UTRs from the Starbase (http://starbase.sysu.edu.cn/starbase2/) and published RBP CLIP-SEQ data sets using IGF2BP3 Enhanced-CLIP SEQ data. We found 16 mRNAs bound to IGF2BP3 (Fig. 7A). Given that hsa_circ_0003258 promotes tumor metastasis, we have identified ten metastasis-related genes as potential targets of hsa_circ_0003258 based on relevant literature reports (Fig. S5A-B). Through qRT-PCR and Western blot validation in DU145 and C4–2 cells, we confirmed that HDAC4 is the target of hsa_circ_0003258 (Fig. 7B-C and S5C). Therefore, we chose HDAC4 for the next research. RIP assay was used to confirm the interaction between IGF2BP3 and HDAC4 in DU145 and C4–2 cells (Fig. 7D and S5D). SRAMP website tools and methylated RNA immunoprecipitation (MeRIP) assay showed HDAC4 was highly enriched in the m6A precipitation part, confirming the m6A modification in HDAC4 (Fig. 7E-F). Western blot analysis showed that enhanced the expression of IGF2BP3 functionally rescued the decreased HDAC4 level upon hsa_circ_0003258 silencing (Fig. 7G).

We further found that knockdown of hsa_circ_0003258 and IGF2BP3 significantly caused the reduction of HDAC4 expression, indicating the reduced stability of the mRNA of HDAC4 (Fig. 7H-K). As reported, HDAC4 induces EMT in glioma cells and contributes to progression of esophageal carcinoma [18, 19]. We further evaluated whether HDAC4 can enhance the metastatic ability in PCa cells. HDAC4 was found significantly up-regulated in PCa cells (Fig. S6A). Transwell assay showed that downregulation of HDAC4 significantly decreased the number of migratory PCa cells (Fig. 7L and S6B). Moreover, Western blot analysis exhibited that depletion of HDAC4 markedly decreased the level of E-cadherin and p-ERK protein, suggesting that hsa_circ_0003258 promotes EMT in PCa cells through inhibiting the HDAC4 and MAPK signaling pathways (Fig. 7M). These findings indicated that hsa_circ_0003258/IGF2BP3 enhances the stability of HDAC4 mRNA by forming RNA-protein ternary complex, thereby promoting the metastasis of PCa. Finally, to confirm whether hsa_circ_0003258 can promote the metastasis of PCa cells through the two signaling pathways, we inhibited the two signaling pathways and found that the metastatic ability of PCa cells reduced much more in the combined inhibition than inhibition of a single pathway alone (Fig. S6C).

### Hsa_circ_0003258 promotes the metastasis of PCa in vivo

**Fig. 8:**
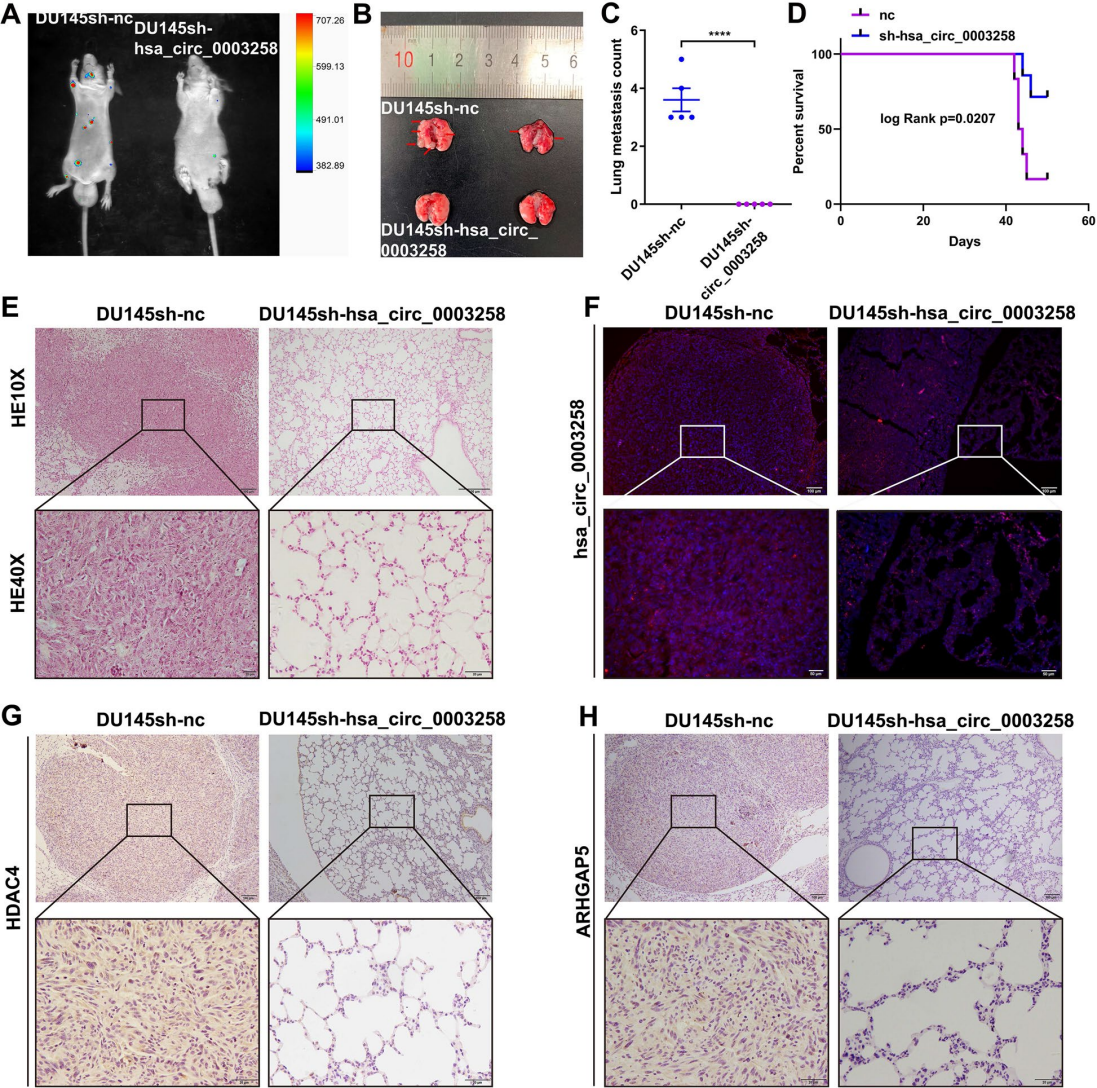
Hsa_circ_0003258 promotes the metastasis of PCa cells in vivo. **A**-**B** The luciferase images and the gross observation of lung metastases in mice injected with DU145/sh-nc and DU145/sh-hsa_circ_0003258 cells (*n* = 5). **C** The number of metastatic nodules in lungs. **D** Kaplan–Meier overall survival curves of mice with PCa stratified by sh-hsa_circ_0003258 knockdown and negative control (log-rank test, n = 5, *P* = 0.0207). **E** The lung sections were stained with H&E. **F** Representative images of FISH analysis of hsa_circ_0003258 in lung tissues. **G**-**H** HDAC4 and ARHGAP5 expression in lung tissues detected by IHC. **** *P* < 0.0001

To evaluate hsa_circ_0003258 in tumor metastasis in vivo, DU145 cells with stably expressing either control or sh-hsa_circ_0003258 were intravenously injected into the tail vein of BALB/c nude mice. Mice were sacrificed and examined for metastatic tumor nodules formed in the lungs. It was found that the mice injected with cells with hsa_circ_0003258 shRNA had less metastatic nodules in the lungs than mice injected with control cells. (Fig. 8A-C and S6D-E). Moreover, we found that knockdown of hsa_circ_0003258 could prolong the survival time of mice compared with the control group (Fig. 8D). The histopathological characteristics of the tumor tissues were shown by H&E staining (Fig. 8E). There was a significant difference in the expression of hsa_circ_0003258 in the lungs, which was detected by FISH (Fig. 8F). Moreover, the IHC results of tumor tissues also showed that sh-hsa_circ_0003258 had lower expression of HDAC4 and ARHGAP5 in the lungs compared with the vector (Fig. 8G-H).

## Discussion

CircRNAs are spliced form pre-mRNAs and play crucial roles in regulating chromatin dynamics and protein expression [20]. Recently, more and more circRNAs have been noticed dysregulated in different kinds of malignancies, which participated in diversified physiological and pathological processes of tumors cells, including proliferation, cell cycle progression, and metastasis [9]. They can be potential diagnostic biomarkers and targets for tumor therapy. However, the functions of most circRNAs are still to be explored for there are just limited experimental evidence reveal the role they played in different kinds of tumors. Liquid biopsy is a non-invasive method that uses body fluids, such as plasma, urine and serum, to detect disease states [21, 22]. Besides, some research has confirmed that circRNAs play critical roles in liquid biopsy [23–26]. They are often detected not only in tumor tissues and cells but also in body fluids from cancer patients. Interestingly, our previously study identified circRNAs from patients’ serum and proved that cirZMIZ1 could facilitate the growth of PCa cells and might act as a liquid biomarker for PCa [14]. This study implied that circRNAs in the body fluids may be promising biomarkers and even potential therapeutic targets for human malignancies, including PCa.

In order to further identify potentially valuable circRNAs in body fluids of PCa patients, circRNAs microarray scanning and bioinformatics analyses were performed on plasma extracted from peripheral blood specimen. Interestingly, we found a novel potential tumor metastasis promoting circRNA termed hsa_circ_0003258. Previously, hsa_circ_0003258 has been reported to promote metastasis of hepatocellular carcinoma by sponging miR-203/miR-502-5p [27]. Furthermore, exosomal circ-0003258 promotes hepatocellular carcinoma progression via miR-29a-3p/GUCD1 Axis [28]. However, whether hsa_circ_0003258 is involved in the development and progression of PCa is still elusive. Here, we identified hsa_circ_0003258 was upregulated in PCa tissues and cells, and which is associated with patient’s clinical parameters. Results of functional assays revealed that ectopic expression of hsa_circ_0003258 significantly increased cell migration. On the contrary, knockdown of hsa_circ_0003258 produced the opposite effects. Finally, through in vivo experiments, we further confirmed that hsa_circ_0003258 could promote the lung homing capacity of PCa cells. Interestingly, our results demonstrated that silencing of hsa_circ_0003258 largely increased the expression of E-cadherin, but not vimentin, which was consistent with previous reports [3, 29]. Notably, changes in epithelial markers and changes in mesenchymal markers do not always occur at the same time during the EMT process, loss of E-cadherin was reckoned as the crucial step to initiate EMT that sustained tumor metastasis. Furthermore, our GO and KEGG analysis showed that the expression of hsa_circ_0003258 was correlated with cell adhesion and focal adhesion. Actually, E-cadherin not only acts as an epithelial marker during EMT, but also knows to be implicated in cell adhesion. It is well known that reduced cell adhesion is considered to be the initiation of the elial-EMT process [30]. Collectively, these results suggested that hsa_circ_0003258 facilitates metastasis of PCa via including the EMT pathway.

Accumulating evidence shown that the mechanisms through which circRNAs exert biological functions are diverse, including serving as microRNA (miRNA) sponge, interacting with RBPs, and even translating proteins. One of the most extensively studies is circRNA terminating the regulation of miRNA to its target gene by binding to the miRNA as a competing endogenous RNA (ceRNA) through the base complementary pairing principle. For instance, CircHIPK3 promotes colorectal cancer via miR-7 [31], circEPSTI1 regulates ovarian cancer via miR-942 [32], circANKS1B regulates breast cancer via miR-148/152 [33] and circ-AMOTL1L regulates PCa via miR-193a-5p [34]. Here, we performed the bioinformatic analysis to predict the potential binding miRNAs of hsa_circ_0003258, and found that miR-653-5p is potentially regulated by hsa_circ_0003258. Then the direct binding relationship between hsa_circ_0003258 and miR-653-5p was validated by RNA pull-down assay and luciferase reporter experiments. Our results showed that hsa_circ_0003258 may act as the sponge for miR-653-5p and inhibit the metastasis of PCa.

Previous studies have shown conflicting results that miR-653-5p may be involved in tumor progression as an oncogene or tumor suppressor. MiR-653-5p has been identified as an oncogene in PCa [35] and gastric cancer [36], whereas acted as a tumor suppressor gene in cervical cancer [37], non-small cell lung cancer [38] and melanoma [39]. On the contrary, we found that miR-653-5p plays a critical tumor suppressor role in the process of inhibiting PCa metastasis by suppressing EMT. It is well known that miRNAs can inhibit translation or reduce mRNA stability by directly targeting gene 3′-UTR, thereby acting as a post-transcriptional regulator. In this study, we explored that hsa_circ_0003258 may function as an endogenous miRNA sponge to inhibit the expression of miR-653-5p by binding to miR-653-5p in PCa cells, leading to diminish the repression of miR-653-5p on ARHGAP5 3′-UTR and eventually facilitate cell metastasis. Moreover, upregulation of miR-653-5p significantly reversed the expression of ARHGAP5 and the increased metastasis induced by overexpression of hsa_circ_0003258. Therefore, these data exhibit that hsa_circ_0003258 partially regulates the stability of ARHGAP5 by acting as a ceRNA in PCa.

The tertiary structure of circRNAs leads to higher protein adsorbing capacity than linear RNA sequences. Thus, the circRNA-interacting RBPs act as an important molecular mode of action in the occurrence, translation, transcription regulation and extracellular transport of target genes [40]. For example, circFOXK2 complexed with YBX1 and hnRNPK to promote PDAC progression [41]. CircFOXP1 contributed to gallbladder cancer progression by interacting with PTBP1 and sponging miR-370 [42]. CircNDUFB2 in complex with IGF2BPs inhibited non-small cell lung cancer progression [43]. Considering that it is a function of circRNA to interact with RBPs, we further explored the potential RBPs of hsa_circ_0003258 to understand its mechanism. Our results demonstrated that hsa_circ_0003258 could directly bind to IGF2BP3 to enhance the stability of HDAC4 mRNA and thereby promote the EMT in PCa. IGF2BP3 is a well-known RBP which prevents mRNA decay by recognizing m6A-modified mRNA [44]. Numerous studies have shown that IGF2BP3 is ubiquitously expressed in eukaryotic tissues and is often up-regulated in human cancers [45]. Previous study showed that IGF2BP3 could form a ternary complex of circFNDC3B-IGF2BP3-CD44 mRNA to increase CD44 expression in the translation process, finally promoting the migration and invasion of gastric cancer cells [46]. Herein, we hypothesized that hsa_circ_0003258 might function its role in PCa metastasis by the same mechanism. RNA immunoprecipitation assays and RNA pull-down assays confirmed that hsa_circ_0003258 increased HDAC4 expression via the formation of a ternary complex of hsa_circ_0003258-IGF2BP3-HDAC4 mRNA, thereby increasing HDAC4 mRNA stability. Intriguingly, knockdown of IGF2BP3 abolished the effects of hsa_circ_0003258 on the protein expression of HDAC4, which indicates HDAC4 is the downstream effector molecule of hsa_circ_0003258. HDAC4, which belongs to class IIa of the HDAC family, may contribute to tumor development and progression through multiple mechanisms [47, 48]. However, its biological roles in the tumor metastasis of PCa remain elusive. Here, our results demonstrated that HDAC4 silencing reduced the cell migration of PCa. After all, from the perspective of circRNA regulation, we have revealed a unique function, that is, circRNAs can interact with RBPs to improve the stability of mRNA.

The Ras-Mitogen-Activated Protein Kinase (MAPK) pathway that comprise of the extracellular signal-related kinase1/2 (ERK1/2), JNK and p38 MAPK which play key roles in regulating multiple cellular processes like cell proliferation, apoptosis and migration [49]. ERK activates the downstream RAS/RAF/MEK/ERK and results in cancer progression [50]. Inhibiting RAS and related GTPases will result inhibition of targeting the downstream effectors of RAS signaling, including the RAF-MAPK/ERK kinase pathway and the PI3K-AKT-mTOR kinase pathway [51, 52]. In this study, we observed that when silencing of hsa_circ_0003258 the differentially expressed genes were enriched in the MAPK signaling pathway. Then our further validation experiments revealed that hsa_circ_0003258 could upregulate the expression of p-ERK. These results suggested that hsa_circ_0003258 could accelerate PCa metastasis through ERK signaling pathway.

However, it is interesting that hsa_circ_0003258 was downregulated in the plasma level, but our results demonstrate that hsa_circ_0003258 was significant up-regulated in PCa cells and tissues. Besides, hsa_circ_0003258 acted as a promising biomarker for metastasis of PCa. These contradictory results between cell and plasma levels suggested that there could be more molecular mechanisms for hsa_circ_0003258 in regulating PCa progression. Based upon, we assumed that hsa_circ_0003258 may be affected by the tumor micro-environment during the process of being released into the plasma, and may be taken up by cells in the micro-environment. As far as we know, plasma is such a mixture of the secreta of the whole-body cells. In addition, some studies focusing on the tumor micro-environment also confirm our hypothesis. For example, Loss of miR-203 promotes tumor growth in some tumors [53] but some evidences show that high expression of exosomal miR-203 was associated with a higher TNM stage in colorectal cancer patients [54]. In addition, it was found that compared with adjacent normal tissues, circRIP2 was reduced in bladder cancer tissues. More CD3/CD8 cells in higher circRIP2 expressed patients were identified when compared with lower circRIP2 expression. These findings suggest that the oncogenic circRIP2 may play a tumor suppressive role by communicating with the micro-environment comprehensively. But circRIP2 can promote bladder cancer progression via inducing EMT by activating miR-1305/Tgf-β2/smad3 pathway in vitro. This inconsistency between clinic and biological level suggests that there could be more molecular mechanisms for circRIP2 in regulating bladder cancer behaviors [55]. Taken together, these results indicate that the involvement of tumor micro-environment must be considered if we want to fully understand the function of hsa_circ_0003258.

## Conclusions

**Fig. 9:**
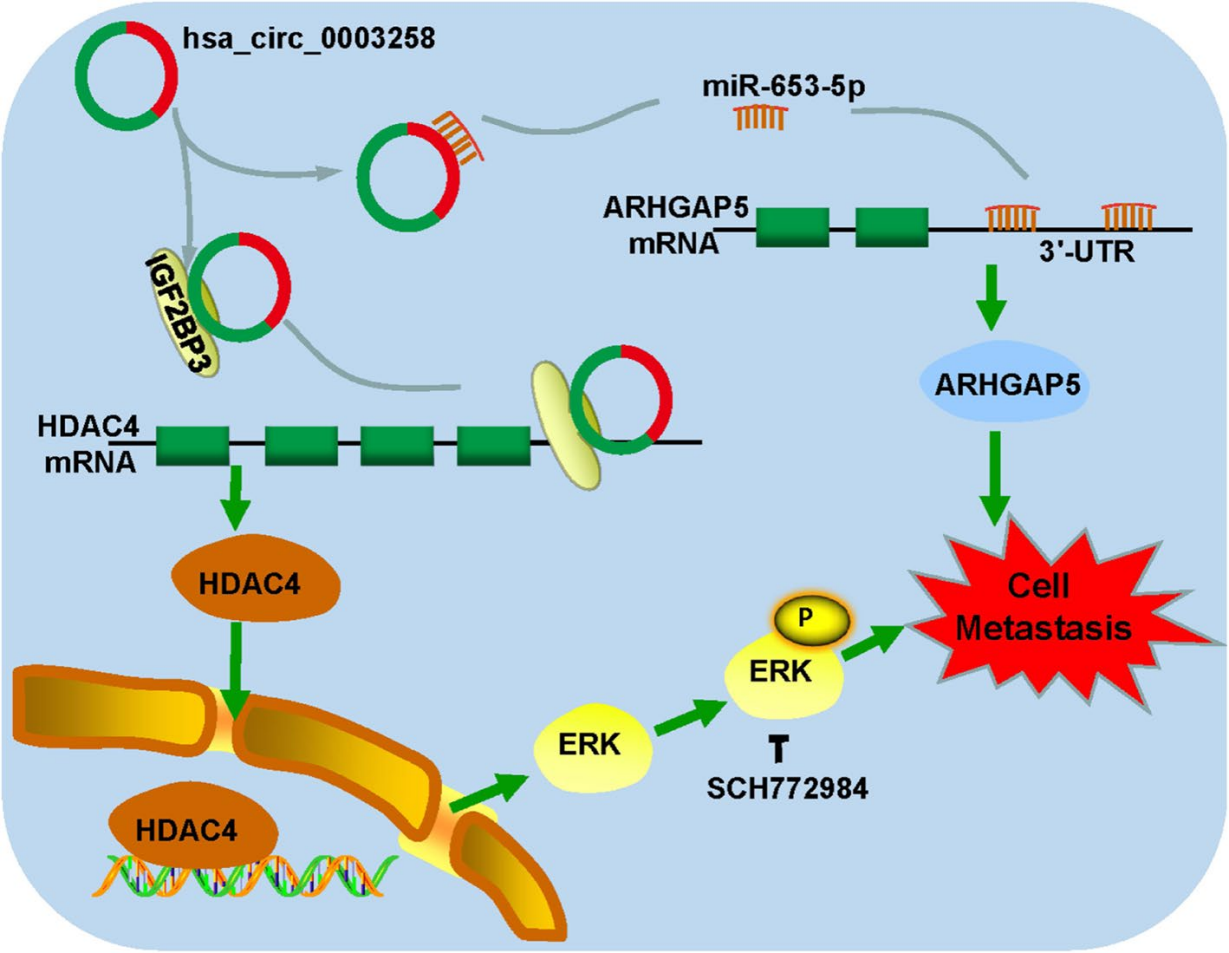
A schematic illustration of the molecular mechanism of hsa_circ_0003258 in promoting the development of PCa

Collectively, our findings suggest that hsa_circ_0003258 functions as a novel positive regulator for PCa metastasis through both hsa_circ_0003258/miR-653-5p/ARHGAP5 axis and hsa_circ_0003258/IGF2BP3/HDAC4 axis (Fig. 9). These findings provide new insights into PCa metastasis and a novel target for PCa treatment.

## Supplementary Information

**Additional file 1: Supplementary Figure 1.** The expression and function of hsa_circ_0003258 in PCa. (A). The heatmap showing some of the differentially expressed circRNAs between normal and prostate cancer patient plasma samples. (B). The representative images of FISH analysis of hsa_circ_0003258 in normal and PCa tissues. (C). The protein coding potential of hsa_circ_0003258. (D). The melting curve of hsa_circ_0003258 amplified product using the divergent primers. (E-H). Transwell assay detecting the migratory capacity of PCa cells after transfecting with siRNAs or vector plasmid. (I-J). Colony formation assays and CCK8 assays were utilized to evaluate the proliferation of PCa cells after knockdown of hsa_circ_0003258 in DU145 and C4–2 cells. ns: Not Significant. **Supplementary Figure 2.** Hsa_circ_0003258/ miR-653-5p/Arhgap5 pathway in C4-2. (A). The heatmap of differentially expressed mRNAs in DU145/sh-nc and DU145/sh- hsa_circ_0003258 cells. Each sample was mixed in three replicates. (B). Transwell assay detecting the migratory capacity of PCa cells after SCH772984 treatment for 48 h in DU145 Lv- hsa_circ_0003258 cells. (C). The potential binding miRNAs predicted by CircInteractome database. (D). Lysates from C4–2 cells was subjected to biotinylated-hsa_circ_0003258 pull-down assay and the expression levels of hsa_circ_0003258 were measured by qRT-PCR. (E). The expression levels of the top three candidate miRNAs predicted by Circinteractome database quantified by qRT-PCR after biotinylated-hsa_circ_00032583 pull-down assay in C4–2 cells. (F). The relative expression of hsa_circ_0003258 detected by qRT-PCR after the transfection of miR-653-5p mimic or inhibitor. (G-H). The qRT-PCR and Western blot showing the ARHGAP5 mRNA and protein level change of the predicated targets after silencing or overexpression of hsa_circ_0003258. (I-J). The qRT-PCR and Western blot showing the ARHGAP5 mRNA and protein level change of the predicated targets after silencing or overexpression of miR-653-5p. ns: Not Significant; * *P* < 0.05; ** *P* < 0.01; *** *P* < 0.001. **Supplementary Figure 3.** MiR-653-5p and ARHGAP5 promote metastasis of PCa. (A). Cell migration observed in transwell assays after overexpression or silencing of miR-653-5p in C4–2 cells. (B). Transwell assay was performed after transfection with indicated vectors, lv- hsa_circ_0003258, mimic-nc or mimic-miR-653-5p in C4–2. (C). Transwell assay detected the migratory capacity of C4–2 cells after silencing ARHGAP5. ns: Not Significant; ** *P* < 0.01; *** *P* < 0.001; **** *P* < 0.0001. **Supplementary Figure 4.** The RNA-binding protein IGF2BP3 binds to hsa_circ_0003258 in C4-2. (A). UP Western blot analysis of IGF2BP3 levels in pulldown assays using a biotinylated antisense oligomer targeting the junction of hsa_circ_0003258 in C4–2. Down RIP assay showing the association of IGF2BP3 with Hsa_circ_0003258in C4–2. Relative enrichment representing RNA levels associated with IGF2BP3 relative to an input control. IgG antibody served as a control. (B). IF and FISH assay showing hsa_circ_0003258 colocalized with IGF2BP3 protein in the cytoplasm in C4–2. (C). The m6A modification site of hsa_circ_0003258 predicted by SRAMP website tools. ** *P* < 0.01. **Supplementary Figure 5.** Hsa_circ_0003258 binds to IGF2BP3 and enhances its interaction with HDAC4. (A-B). Flow chart illustrates the criteria of identifying of HDAC4 as the target of hsa_circ_0003258. (C). The qRT-PCR showing the mRNA level change of the predicated targets after silencing of hsa_circ_0003258 in C4–2 cells. (D). RIP assay showing the association of IGF2BP3 with HDAC4 in C4–2 cells. Relative enrichment representing RNA levels associated with HDAC4 compared to an input control. IgG antibody served as a control. ns: Not Significant; * *P* < 0.05; ** *P* < 0.01; ***P < 0.001. **Supplementary Figure 6.** The expression and function of HDAC4 in PCa cells and the images of lung metastasesin mice. (A). HDAC4 protein and mRNA expression in the normal prostate epithelial cell line (RWPE-1) and five PCa cell lines detected by Western blot and qRT-PCR. (B). Transwell assay detected the migratory capacity of C4–2 cells after silencing HDAC4. (C). Transwell assay detected the migratory capacity after silencing the two signaling pathways. (D). Images of lungs derived from DU145 cells transfected with nc and sh- hsa_circ_0003258. (E). The luciferase images and the gross observation of lung metastases in mice injected with DU145/sh-nc and DU145/sh-hsa_circ_0003258 cells (*n* = 5). * *P* < 0.05; ** *P* < 0.01; *** *P* < 0.001.

**Additional file 2: Supplementary Table S1.** Oligos used in the study.

**Additional file 3: Supplementary Table S2**. The detailed clinicopathological data of enrolled patient.

## Data Availability

The datasets used and/or analyzed during the current study are available within the manuscript and its supplementary information files.

## Abbreviations

CircRNA: Circular RNAs
PCa: Prostate cancer
qRT-PCR: Quantitative real-time polymerase chain reaction
ceRNA: Competing endogenous RNA
ISUP: Internal Society of Urological Pathology
EMT: Epithelial to mesenchymal transition
FISH: Fluorescence in situ hybridization
H&E: Hematoxylin and eosin
IHC: Immunohistochemistry
RBP: RNA binding protein
ARHGAP5: Rho GTPase activating protein 5
IGF2BP3: Insulin like growth factor 2 mRNA binding protein 3
HDAC4: Histone deacetylase 4
