# Supplementary material for: Hsa_circ_0003258 promotes prostate cancer metastasis by complexing with IGF2BP3 and sponging miR-653-5p
Source: Mol Cancer. 2022 Jan 5;21:12. doi: 10.1186/s12943-021-01480-x (PMC8729084; doi:10.1186/s12943-021-01480-x)
Supplement: Supplementary file 2 — Additional file 2: Supplementary Table S1. Oligos used in the study. [file 12943_2021_1480_MOESM2_ESM.docx]

Supplementary Material

# 1 SupplementaryTables

# 1.2 Supplementary Tables

**Supplementary Table S1. Oligos used in the study.**

| **Name** | **Sequence** |
| --- | --- |
| hsa_circ_0003258 junction probe | /5bio/-GTCTTTTGTGTGTGCAGTGT |
|  | /5bio/-ATGTCTTTTGTGTGTGCAGT |
|  | /5bio/-ATGGCATGTCTTTTGTGTGT |
| hsa_circ_0003258 control probe | /5bio/-TGTTGTTCTTGGTCTGTAGG |
|  | /5bio/-GATTGGTTGTCTGTACTGTT |
|  | /5bio/-TATGTTGTTGATTTGCTGGC |
| si-h-hsa_circ_0003258_001 | ACACACTGCACACACAAAA |
| si-h-hsa_circ_0003258_002 | ACTGCACACACAAAAGACA |
| si-IGF2BP3-1 | GTGGACTCGTCCAAGATCA |
| si-IGF2BP3-2 | CGATGACTTCGATCTGGAA |
| si-ARHGAP5-1 | GTACGAATTTGCAACCATA |
| si-ARHGAP5-2 | GAAGGACTCTACCGTGTCA |
| si-HDAC4-1 | CGACAGGCCTCGTGTATGA |
| si-HDAC4-2 | CCACGAGCACATCAAGCAA |
| ZNF652-F | GCTGGTTGAAAACTGTGCTGT |
| ZNF652-R | GAAGATGGCACTTGACCACGA |
| hsa_circ_0003258-F | CACATGAAAACACACACTGCAC |
| hsa_circ_0003258-R | TCGTCACAGTTCTCGCATCT |
| 18SrRNA-F | ACACGGACAGGATTGACAGA |
| 18SrRNA-R | GGACATCTAAGGGCATCACA |
| U6-F | CTCGCTTCGGCAGCACA |
| U6-R | AACGCTTCACGAATTTGCGT |
| GAPDH-F | CAGTCAGCCGCATCTTCTT |
| GAPDH-R | GACAAGCTTCCCGTTCTCAG |
| IGF2BP3-F | TATATCGGAAACCTCAGCGAGA |
| IGF2BP3-R | GGACCGAGTGCTCAACTTCT |
| RAI14-F | AGCCCAAGATACTACCGGACA |
| RAI14-R | CGCTGCATAATGTAAAGCTGTTT |
| UBN1-F | CCTGAATCCTGCGTTTTTGAAG |
| UBN1-R | GCAGCGTTTGTGATCTGGTT |
| RSF1-F | CCAGCAAGGTCAACCTCGT |
| RSF1-R | CAGAGCCGTTGTACTGAATGT |
| ARHGAP5-F | AGGGAAGCTCAACGTAGATGG |
| ARHGAP5-R | ATGATCCACGCATTCATCACAT |
| ZBTB10-F | CGGCTCCACGAACAATAACG |
| ZBTB10-R | CAGGCCCTCCAATTCCACTT |
| ATXN1-F | CTGACAGTGGACCAGTATGGC |
| ATXN1-R | GGGCAAGGGACTCATATTCAC |
| EIF3A-F | GCAAAGGAGGGGTTATACCAGT |
| EIF3A-R | TGTCAGTACGATCCTGAGTGTC |
| SCHIP1-F | ACTCTGACAGTGATTACCGAGA |
| SCHIP1-R | GGAAGGGCTCCTCATAGTCC |
| NPTX1-F | CACCGAGGAGAGGGTCAAGAT |
| NPTX1-R | CAGGGCGGTTGTCTTTCTGA |
| SPOCK1-F | CCCAACCACGGCAATTTCCTA |
| SPOCK1-R | ATCGTCTCGAAAGCGGTTCC |
| OGT-F | TCCTGATTTGTACTGTGTTCGC |
| OGT-R | AAGCTACTGCAAAGTTCGGTT |
| ZEB2-F | GGAGACGAGTCCAGCTAGTGT |
| ZEB2-R | CCACTCCACCCTCCCTTATTTC |
| CDH2-f | TCAGGCGTCTGTAGAGGCTT |
| CDH2-R | ATGCACATCCTTCGATAAGACTG |
| VCAN-F | GTAACCCATGCGCTACATAAAGT |
| VCAN-R | GGCAAAGTAGGCATCGTTGAAA |
| CREB5-F | AAAGACTGCCCAATAACAGCC |
| CREB5-R | AAGCTGGGACAGGACTAGCA |
| HDAC4-F | AGCGTCCGTTGGATGTCAC |
| HDAC4-R | CCTTCTCGTGCCACAAGTCT |
| SULF2-F | TTGCCGTGTACCTCAATAGCA |
| SULF2-R | TTCCGACACAGCGTGTAGTTA |
| EYA4-F | CTTCTTGCAGTCAAAACAGAGC |
| EYA4-R | GTGGATAGGGCTTGGAAGGAT |
