# Supplementary material for: Hsa_circ_0003258 promotes prostate cancer metastasis by complexing with IGF2BP3 and sponging miR-653-5p
Source: Mol Cancer. 2022 Jan 5;21:12. doi: 10.1186/s12943-021-01480-x (PMC8729084; doi:10.1186/s12943-021-01480-x)
Supplement: Supplementary file 1 — Additional file 1: Supplementary Figure 1. The expression and function of hsa_circ_0003258 in PCa. (A). The heatmap showing some of the differentially expressed circRNAs between normal and prostate cancer patient plasma samples. (B). The representative images of FISH analysis of hsa_circ_0003258 in normal and PCa tissues. (C). The protein coding potential of hsa_circ_0003258. (D). The melting curve of hsa_circ_0003258 amplified product using the divergent primers. (E-H). Transwell assay detecting the migratory capacity of PCa cells after transfecting with siRNAs or vector plasmid. (I-J). Colony formation assays and CCK8 assays were utilized to evaluate the proliferation of PCa cells after knockdown of hsa_circ_0003258 in DU145 and C4–2 cells. ns: Not Significant. Supplementary Figure 2. Hsa_circ_0003258/ miR-653-5p/Arhgap5 pathway in C4-2. (A). The heatmap of differentially expressed mRNAs in DU145/sh-nc and DU145/sh- hsa_circ_0003258 cells. Each sample was mixed in three replicates. (B). Transwell assay detecting the migratory capacity of PCa cells after SCH772984 treatment for 48 h in DU145 Lv- hsa_circ_0003258 cells. (C). The potential binding miRNAs predicted by CircInteractome database. (D). Lysates from C4–2 cells was subjected to biotinylated-hsa_circ_0003258 pull-down assay and the expression levels of hsa_circ_0003258 were measured by qRT-PCR. (E). The expression levels of the top three candidate miRNAs predicted by Circinteractome database quantified by qRT-PCR after biotinylated-hsa_circ_00032583 pull-down assay in C4–2 cells. (F). The relative expression of hsa_circ_0003258 detected by qRT-PCR after the transfection of miR-653-5p mimic or inhibitor. (G-H). The qRT-PCR and Western blot showing the ARHGAP5 mRNA and protein level change of the predicated targets after silencing or overexpression of hsa_circ_0003258. (I-J). The qRT-PCR and Western blot showing the ARHGAP5 mRNA and protein level change of the predicated targets after silencing or overexpressi [file 12943_2021_1480_MOESM1_ESM.pdf]

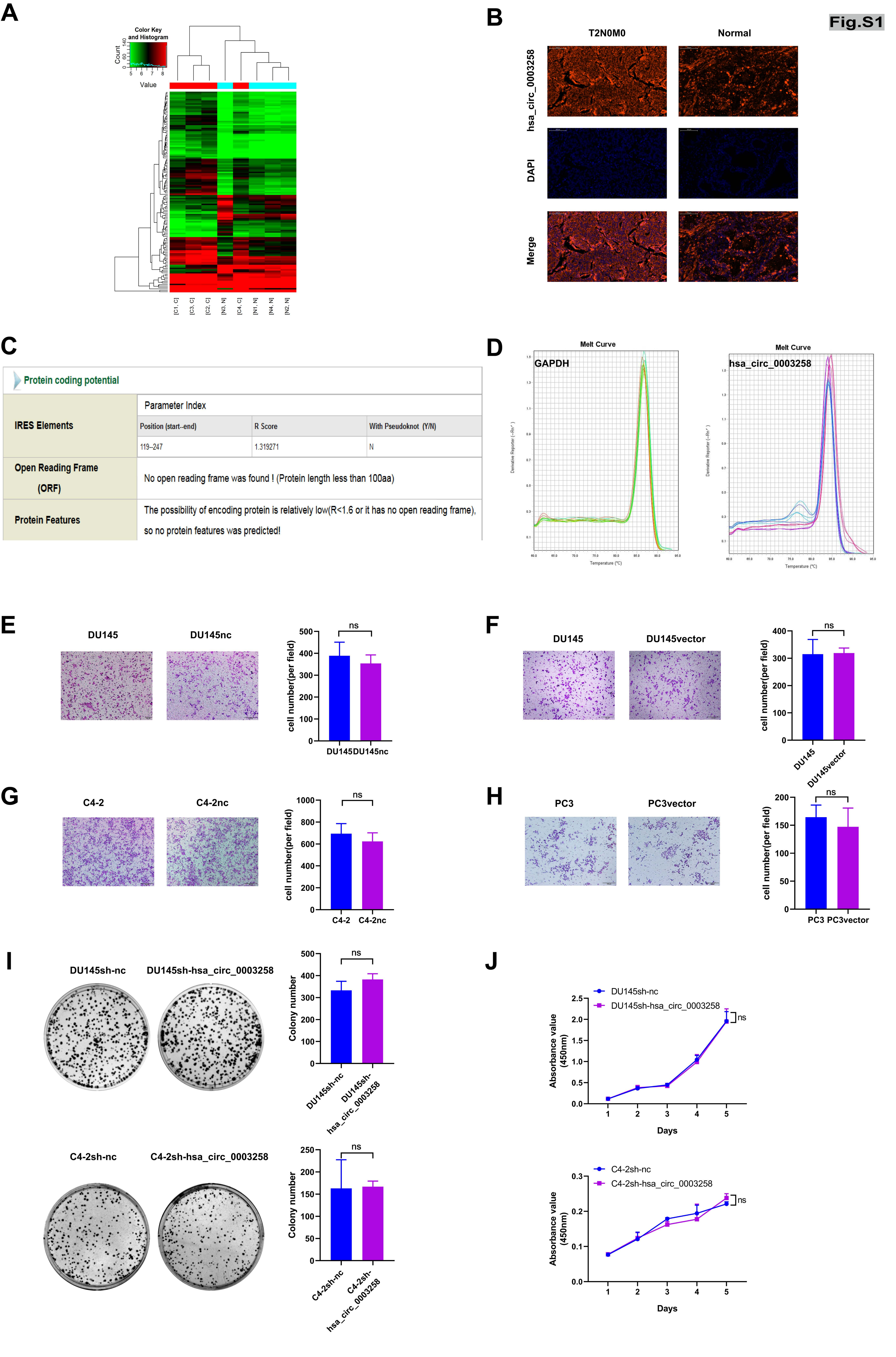

IRES Elements	Parameter Index		
	Position (start--end)	R Score	With Pseudoknot (Y/N)
	119--247	1.319271	N
Open Reading Frame (ORF)	No open reading frame was found ! (Protein length less than 100aa)		
Protein Features	The possibility of encoding protein is relatively low(R<1.6 or it has no open reading frame), so no protein features was predicted!		

Fig.S2

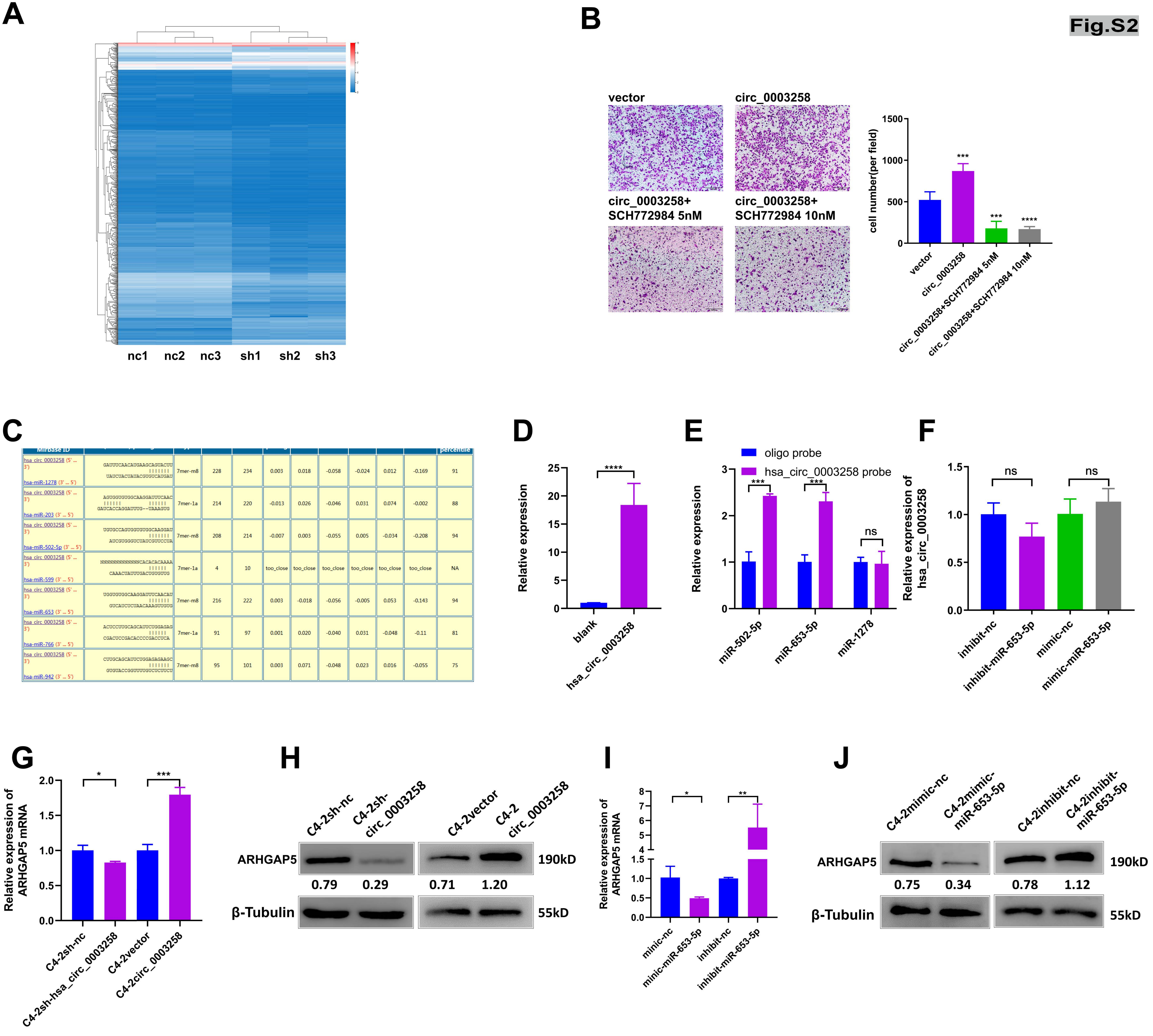

A
nc1
nc2
nc3
sh1
sh2
sh3
B
vector
circ_0003258
circ_0003258+ SCH772984 5nM
circ_0003258+ SCH772984 10nM
cell number(per field)
1500
1000
500
0
***
***
****
vector
circ_0003258
circ_0003258+SCH772984 5nM
circ_0003258+SCH772984 10nM
C
D
Relative expression
****
blank
hsa_circ_0003258
E
oligo probe
hsa_circ_0003258 probe
Relative expression
***
***
ns
miR-502-5p
miR-653-5p
miR-1278
F
Relative expression of hsa_circ_0003258
ns
ns
inhibit-nc
inhibit-miR-653-5p
mimic-nc
mimic-miR-653-5p
G
Relative expression of ARHGAP5 mRNA
*
***
C4-2sh-nc
C4-2sh-hsa_circ_0003258
C4-2vector
C4-2circ_0003258
H
C4-2sh-nc
C4-2sh-circ_0003258
C4-2vector
C4-2 circ_0003258
ARHGAP5
190kD
0.79
0.29
0.71
1.20
β-Tubulin
55kD
I
Relative expression of ARHGAP5 mRNA
*
**
minic-nc
minic-miR-653-5p
inhibit-nc
inhibit-miR-653-5p
J
C4-2mimic-nc
C4-2mimic-miR-653-5p
C4-2inhibit-nc
C4-2inhibit-miR-653-5p
ARHGAP5
190kD
0.75
0.34
0.78
1.12
β-Tubulin
55kD

Fig.S3

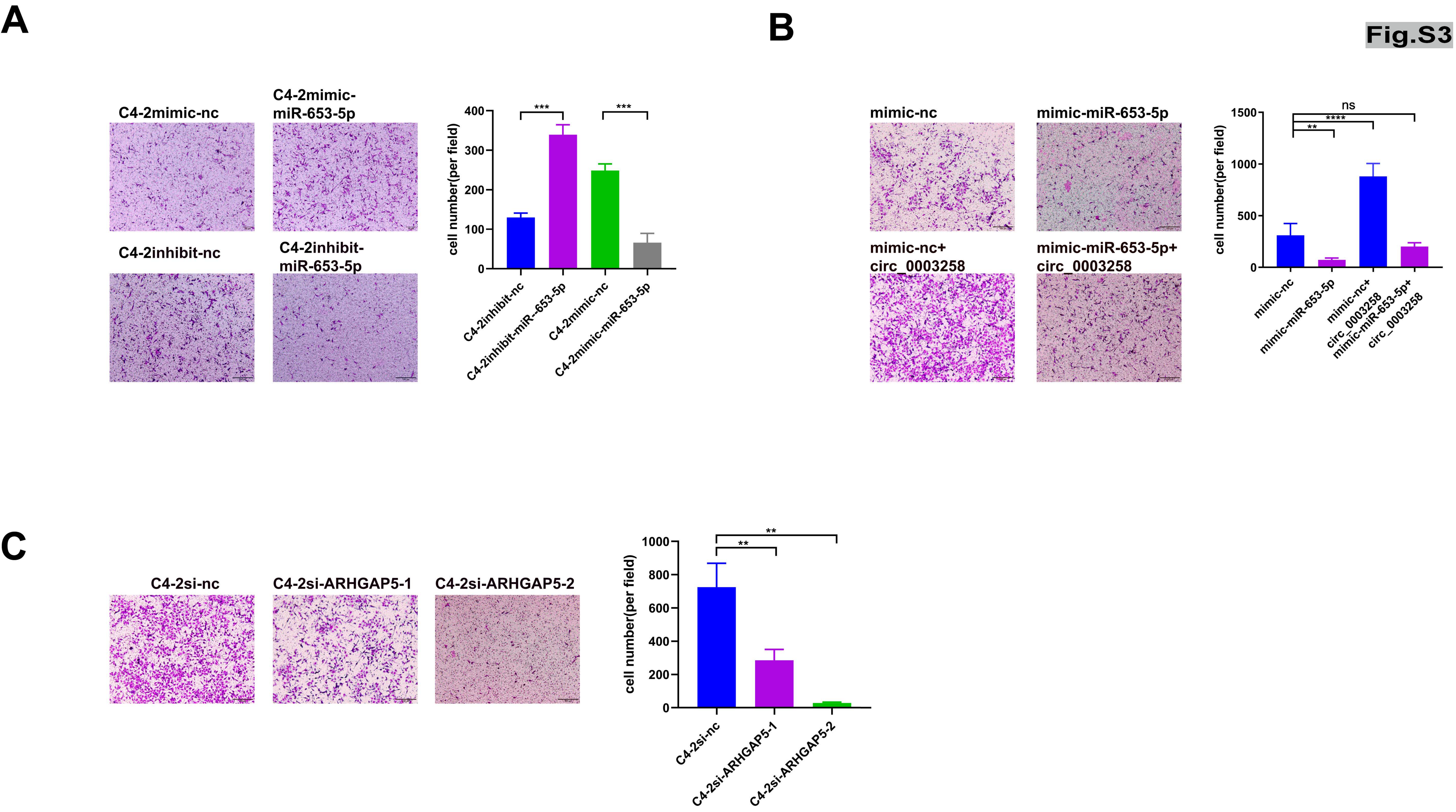
A
C4-2mimic-nc
C4-2mimic-miR-653-5p
C4-2inhibit-nc
C4-2inhibit-miR-653-5p
cell number(per field)
0
100
200
300
400
***
***
C4-2inhibit-nc
C4-2inhibit-miR--653-5p
C4-2mimic-nc
C4-2mimic-miR-653-5p
B
mimic-nc
mimic-miR-653-5p
mimic-nc+ circ_0003258
mimic-miR-653-5p+ circ_0003258
cell number(per field)
0
500
1000
1500
ns
****
**
mimic-nc
mimic-miR-653-5p
mimic-nc+ circ_0003258
mimic-miR-653-5p+ circ_0003258
C
C4-2si-nc
C4-2si-ARHGAP5-1
C4-2si-ARHGAP5-2
cell number(per field)
0
200
400
600
800
1000
**
**
C4-2si-nc
C4-2si-ARHGAP5-1
C4-2si-ARHGAP5-2

Fig.S4

A

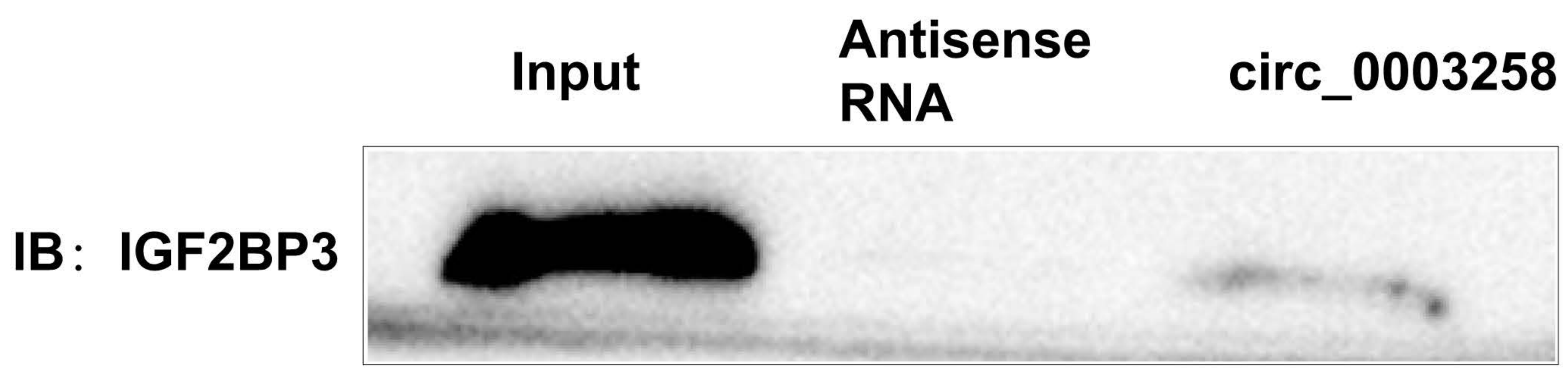

Input
Antisense RNA
circ_0003258
IB: IGF2BP3

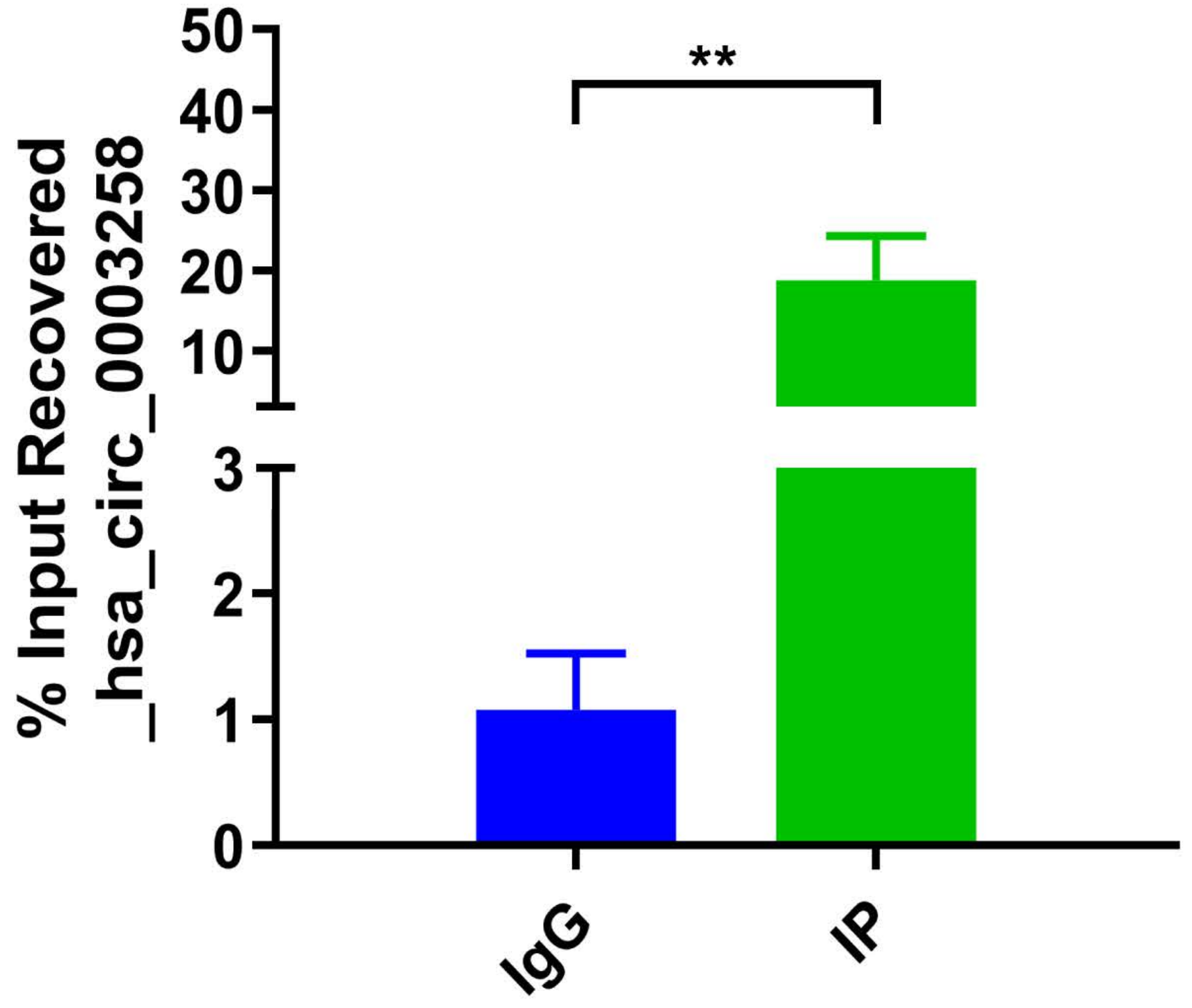

% Input Recovered _hsa_circ_0003258
**
IgG
IP
50
40
30
20
10
3
2
1
0

B

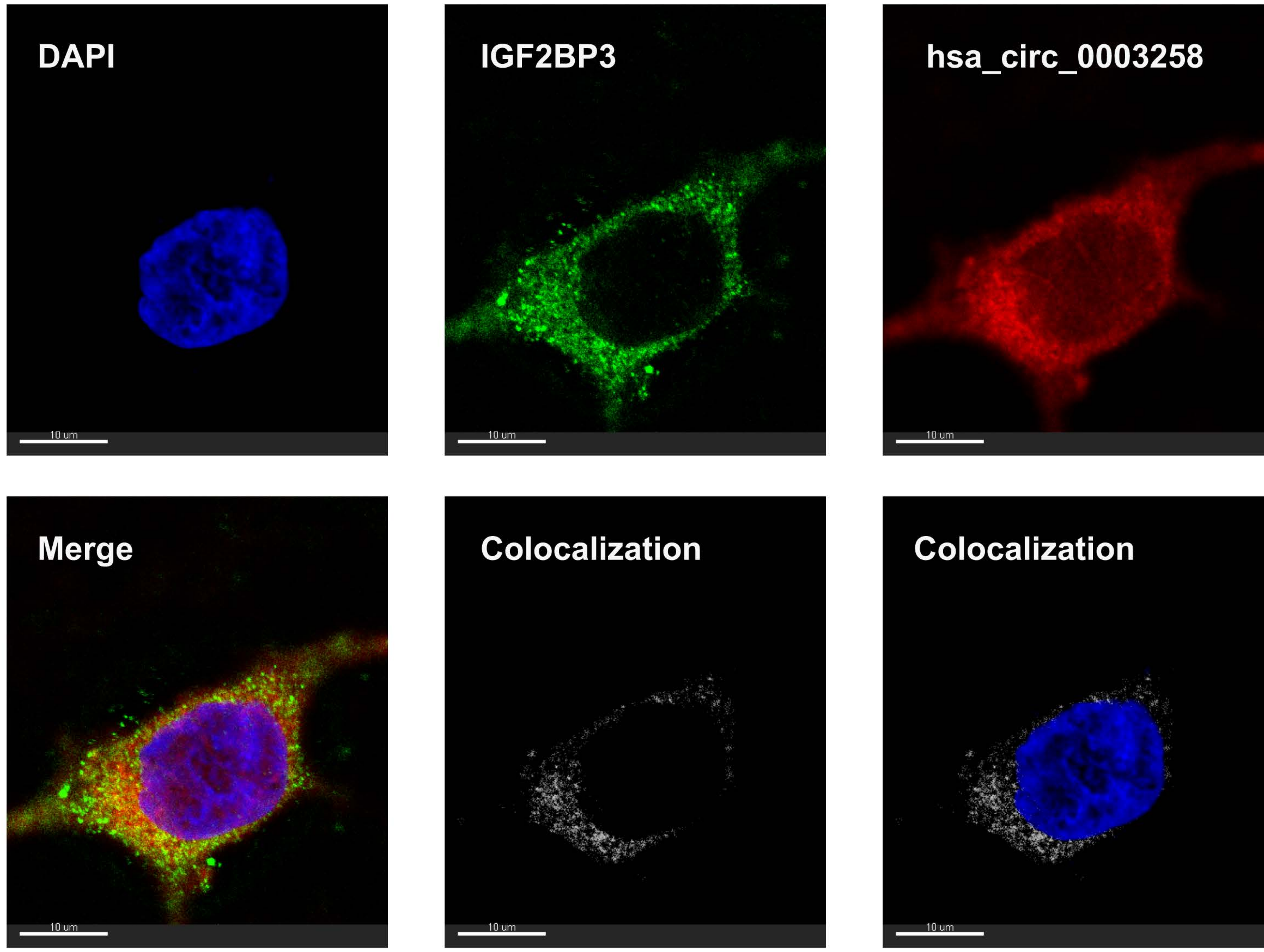

DAPI
IGF2BP3
hsa_circ_0003258
Merge
Colocalization
Colocalization

C

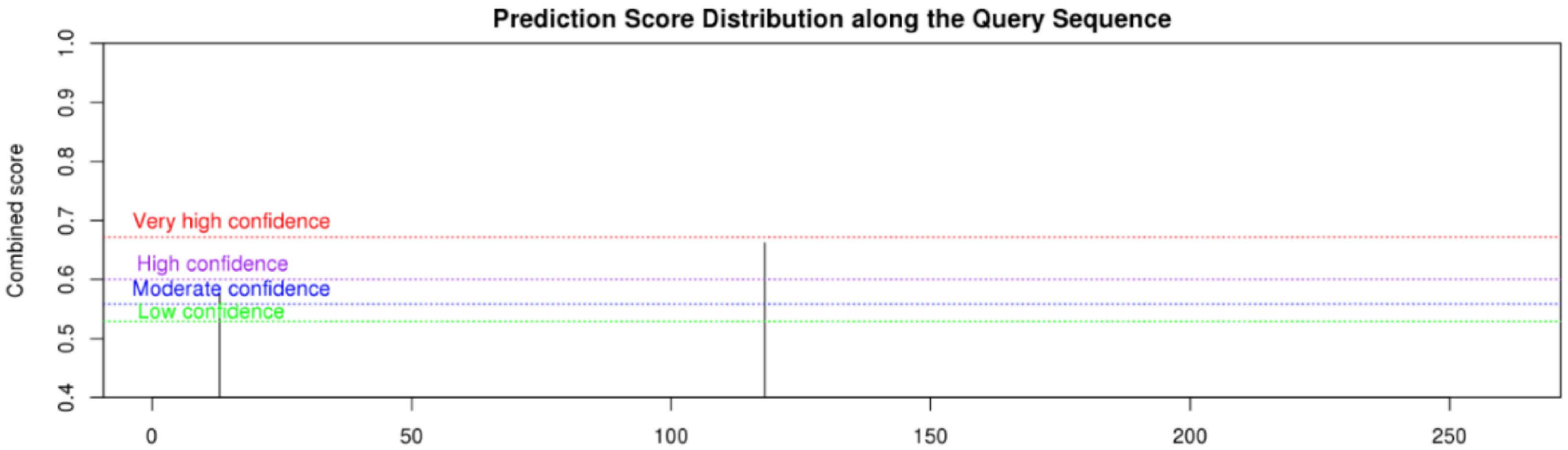

Prediction Score Distribution along the Query Sequence
Combined score
Very high confidence
High confidence
Moderate confidence
Low confidence
1.0
0.9
0.8
0.7
0.6
0.5
0.4
0
50
100
150
200
250

Fig.S5

A

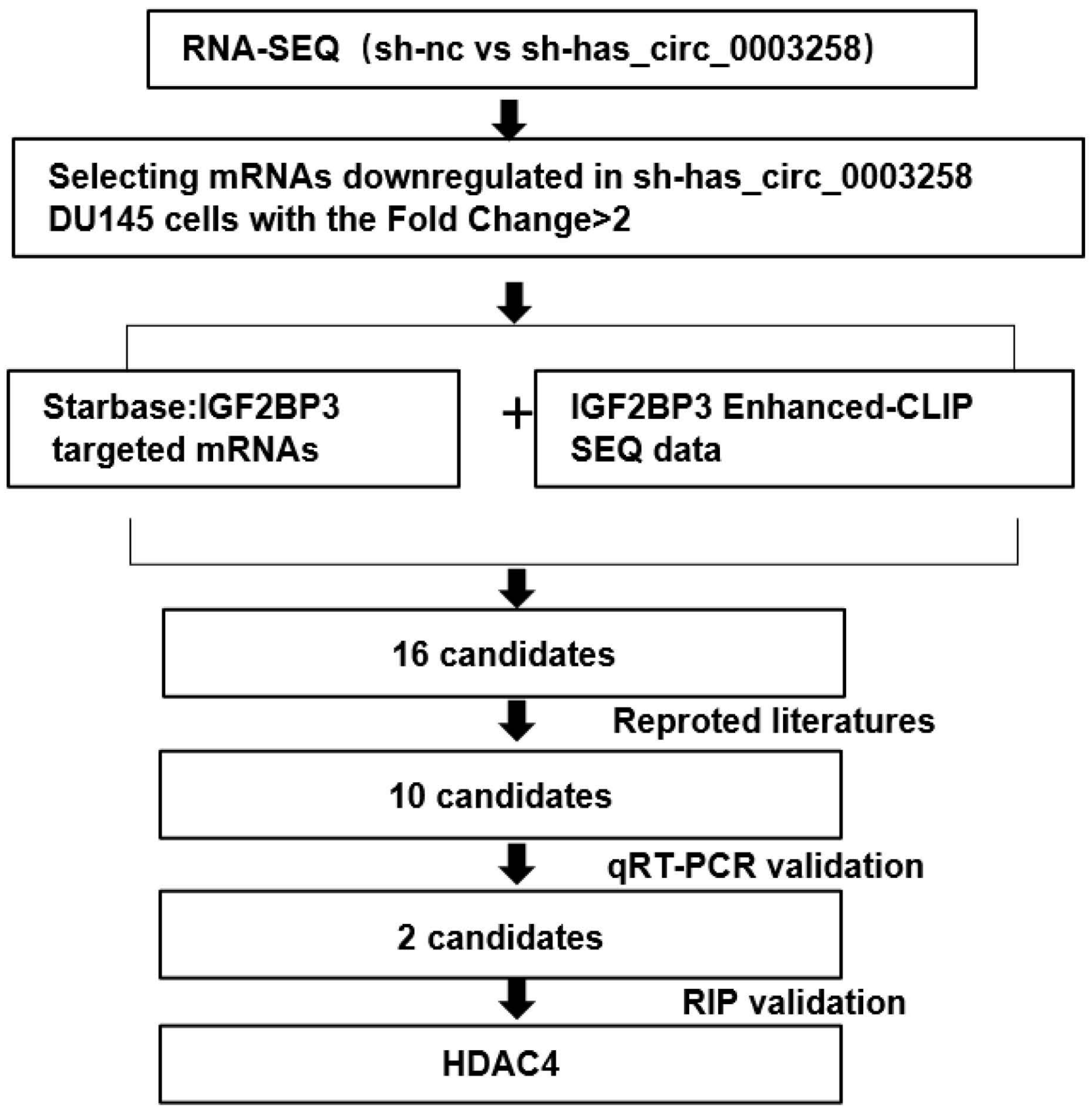

B

Characteristics	Gene Name
Genes downregulated in has_circ_0003258 knockdown PC cells with Fold Change>2.0 and bound by IGF2BP3	VCAN、PHACTR2、NPTX1、PELI2、CREB5、EYA4、CDH2、OGT、ZEB2、SAMD11、HDAC4、SPOCK1、RUNX1T1、CCND1、SULF2、PHF21B
Genes associated with metastasis	SULF2、VCAN、ZEB2、CDH2、SPOCK1、EYA4、NPTX1、HDAC4、CREB5、OGT

C

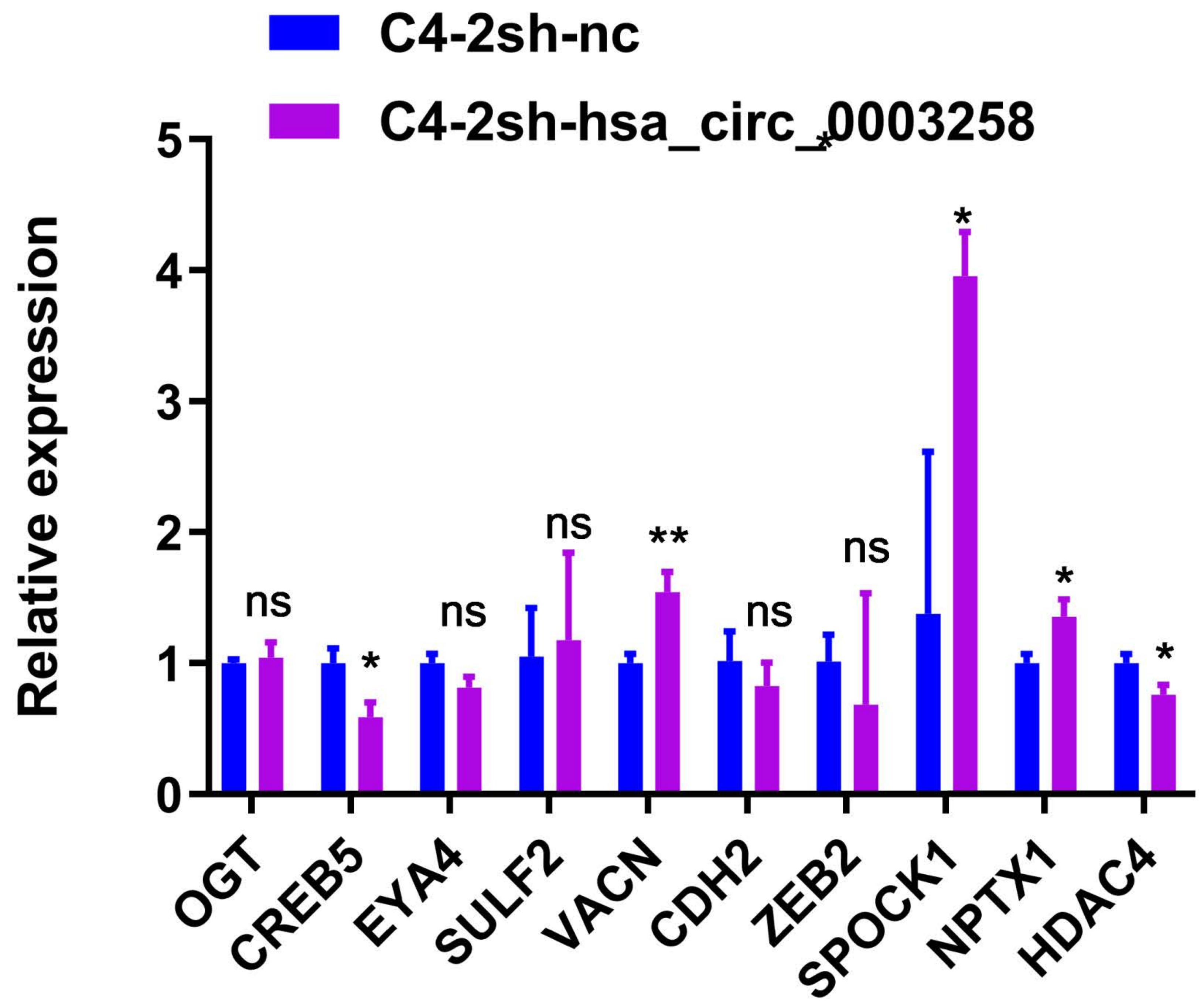

D

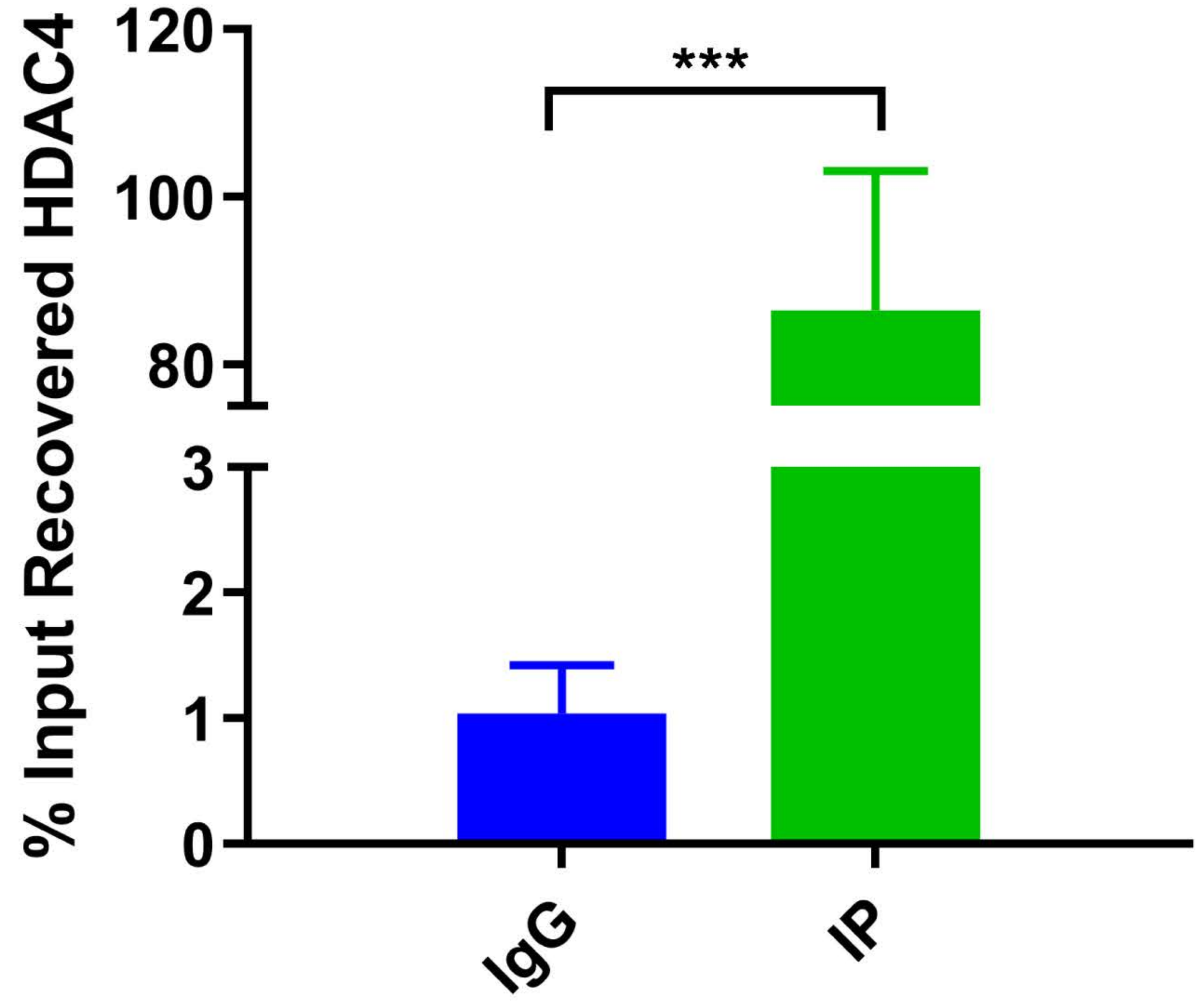

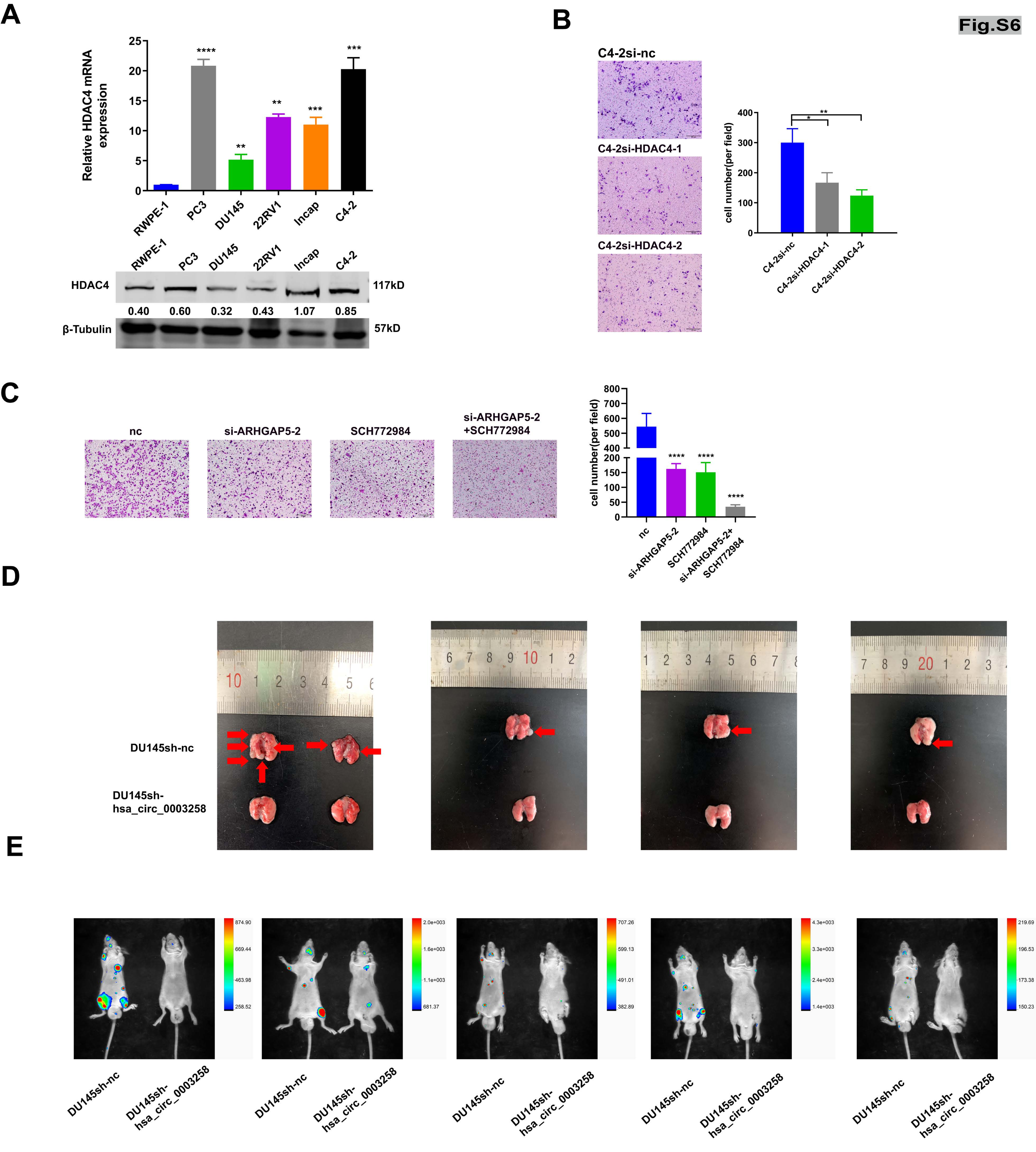
Fig.S6
A
Relative HDAC4 mRNA expression
****
**
**
***
***
RWPE-1
PC3
DU145
22RV1
lncap
C4-2
HDAC4
117kD
0.40
0.60
0.32
0.43
1.07
0.85
β-Tubulin
57kD
B
C4-2si-nc
C4-2si-HDAC4-1
C4-2si-HDAC4-2
cell number(per field)
C
nc
si-ARHGAP5-2
SCH772984
si-ARHGAP5-2 +SCH772984
cell number(per field)
si-ARHGAP5-2+ SCH772984
D
DU145sh-nc
DU145sh-hsa_circ_0003258
E
DU145sh-nc
DU145sh-hsa_circ_0003258
